# Supplementary material for: An initiative for a more inclusive working life and its effect on return-to-work after sickness absence: a multistate longitudinal cohort study
Source: BMJ Open. 2022 Nov 22;12(11):e062558. doi: 10.1136/bmjopen-2022-062558 (PMC9685245; doi:10.1136/bmjopen-2022-062558)
Supplement: Supplementary data [file bmjopen-2022-062558supp001.pdf]

An initiative for a more inclusive working life and its effect on return-to-work after sickness absence – a multi-state longitudinal cohort study

Supplementary Material

S.1 Estimands and statistical methods

A multi-state model can be seen as a stochastic process $\{X(t) \mid t \geq 0\}$, taking values from a set of possible states $S = \{1, 2, 3, ..., S\}$ [1, 2, 3]. In the present study, S = 6, and *t* was days since inclusion. The model is governed by transition intensities $\alpha_{jk}(t)$, referring to the probability of a jump to state *k* from state *j* within a small time interval Δt, divided by Δt, as Δt approaches 0:

$$\alpha_{jk}(t) = \lim_{\Delta t \to 0} \frac{\Pr(X(t + \Delta t) = k \mid X(t -) = j)}{\Delta t}$$

for all j,k in S, where $j \neq k$.

When $\boldsymbol{A}(t)$ is the matrix of cumulative transition intensities with elements $A_{jk}(t) = \int_0^t \alpha_{jk}(s)\, ds$, for $j \neq k$ and $A_{jj}(t) = -\sum_{j \neq k} A_{jk}(t)$, the state probabilities $\boldsymbol{\pi}(t)$ with elements $\pi_j(t) = \Pr(X(t) = j)$ are found using the Aalen-Johansen estimator [4], by

$$\hat{\boldsymbol{\pi}}(t) = \boldsymbol{\pi}(0) \prod_{(0,t]} (\mathbf{I} + \Delta \boldsymbol{A}(u)) \tag{1}$$

where $\boldsymbol{\pi}(0)$ is the initial state distribution at time zero. In settings with time-to-event data, right censoring and no covariates, the elements of $\boldsymbol{A}(t)$ can be estimated using the Nelson-Aalen estimator [4]. State probabilities (also known as state occupational probabilities) are a simple and intuitive quantities for multi-state outcomes and can be seen as an extension of regular survival probabilities (e.g., from Kaplan-Meier curves).

To assess the effect of the Norwegian Inclusive Working Life Agreement (IA), and the within measures for preventing and reducing SA, we sought to identify the average treatment effect (ATE), as the difference in state probabilities

$$\theta(t) = \pi_j^1(t) - \pi_j^0(t) \tag{2}$$

for all $j \in S$, where $\boldsymbol{\pi}^a(t)$ are the counterfactual state probabilities if everyone in the study population was fixed to IA status *a* (taking the values 1 and 0 for IA and non-IA respectively).

From the state probabilities we calculated

$$\theta = \int_0^{365} \pi_j^1(t)\, dt - \int_0^{365} \pi_j^0(t)\, dt \tag{3}$$

representing the difference in expected length of stay (ELOS) in state j, over the first year, for the situations where everyone was fixed to $a = 1$ and $a = 0$.

Hoff R, *et al. BMJ Open* 2022; 12:e062558. doi: 10.1136/bmjopen-2022-062558

The estimands in formula (2) and (3) can be identified under conditional exchangeability (no unmeasured confounding), positivity and consistency [5]. Adjustment for confounding was made by applying baseline inverse probability of treatment (IPT) weights [5], also known as propensity score weights, while fitting hazard models for every possible transition in the model.

More precisely, we fitted a weighted additive hazards regression model [4] on the form

$$\alpha_{jk}(t\,|a) = \beta_{jk0}(t) + \boldsymbol{\beta}_{jk}^{T}(\mathrm{t})a$$

for all possible transitions *jk*, where $\boldsymbol{\beta}^{\mathrm{T}}$ (t) are time-varying regression functions and $\beta_{jk0}(t)$ is the baseline j → k transition intensity. For each observation we applied the baseline inverse probability of treatment (IPT) weight $\widehat{w}_i$, for the corresponding individual *i*. Estimates $\widehat{\boldsymbol{B}}_{jk}(t)$of the cumulative regression functions

$$\boldsymbol{B}_{jk}(t) = \left[\int_0^t \beta_{jk0}(u)du\,, \int_0^t \beta_{jk}^{T}(u)du\right]^T$$

are found using least squares techniques [4]. For the two possible values of a, we then found the cumulative transition intensity matrices by

$$\hat{A}_{jk}^{a}(t) = \hat{A}_{jk}(t|a) = \widehat{\boldsymbol{B}}_{jk}^{T}(t)\boldsymbol{a}^{*}$$

where $\boldsymbol{a}^{*} = (1, a)^{T}$, which again was plugged into the formula in (1), and thus identify the estimands in formula (2) and (3).

To calculate the IPT weights $\widehat{w}_i$, we modelled IA status A, given baseline confounders **Z** in a logistic regression model

$$logit\,\Pr(A|\,\mathbf{Z}) = \beta^{T}\mathbf{Z}$$

Using this model, for each individual *i*, the conditional probability of having their assigned IA status a_i given their covariate values z_i at baseline, $\Pr(a_i \mid \boldsymbol{z}_i)$ were estimated and used to calculate the (stabilized) IPT weight [5]

$$\widehat{w}_i = \frac{\Pr(a_i)}{\Pr(a_i\,|\boldsymbol{z}_i)}$$

where $\Pr(a_i)$ is the sample proportion with IA status equal to a_i at baseline.

S.2 Sensitivity analysis 1: Using subsets of adjustment variables

As a first sensitivity analysis, we compared the results from the main analysis with results from a series of analyses adjusting for all possible subsets of the main adjustment set, including the empty set – in total 256 models. Results are shown in Supplementary Figure 1.

In the left panel of Supplementary Figure 1, the unadjusted differences in state probabilities are seen to be almost twice as big as after confounder adjustment using IPT weighting. While in the right panel, we see how including smaller and less complete sets of confounders can lead to shifts in both directions compared to the main model.

S.3 Sensitivity analysis 2: Adjusting for additional covariates measured for men during military

Most of the men included in the study underwent military conscript examinations at the age of 18. Here they measured, among other things, IQ score, BMI, physical stamina score and a rough assessment of military eligibility (physical and mental). These data were available to us from the Norwegian Armed Forces Personnel Data Base. As these variables were not measured for all the individuals in the cohort, especially women, they were not adjusted for in the primary analyses. However, as a sensitivity analysis, we compared weighed analysis for men not taking and taking these variables into account. The results are shown in the left panel in Supplementary Figure 2. The plot shows no visible differences. In fact, the absolute differences in effect estimates came after the third – fourth decimal. We also tried adding conscript variables in an alternative analysis where the variables for education and workplace industry from the main analysis were left out. Here we were able to detect visible differences in effect estimates from the plot. Results of this analysis are shown in the right panel of Supplementary Figure 2.

S.4 Sensitivity analysis 3: Pre IA (1997-1999) multi-state histories as a negative control

Individual multi-state histories are also available for individuals in the cohort before IA was introduced and can be utilised as a negative outcome control [6]. The question of interest is then whether these earlier multi-state histories differed between those with later IA and those without, and if the IPT weights used sufficiently adjusted for such a difference. We therefore considered state histories during the period from 1st of January 1997 to 31st of December 1999, where we compared both the crude difference in state probabilities for the same intervention groups as in the main analysis, and a weighted analysis using the IPT weights of the main analysis. The results are found in Supplementary Figure 3. Results showed that the later IA groups differed between 1997 and 2000 in terms of education probability (IA group about 4 percentage points more likely to be in education) and work probability (non-IA group about 3 percentage points more likely to be in work). For non-employment there were only small differences. The probabilities for full-time or graded SA did not differ much, as one would expect due to the restriction of no history of SA one year before inclusion. However, after applying the IPT weights from the main analysis, also the differences in work histories that were there disappeared. This indicates that the confounding adjustment through these weights sufficiently covered differences in earlier work life (all states) histories.

S.5 Sensitivity analysis 4: Analysis of a limited selection of industries

From the information in Table 2 in the main article, it appeared that a large proportion of IA companies were in industries where public workplaces are typically overrepresented, such as education and health services. Information on public vs private workplace was not available for the study. In this section, we performed a separate analysis for a limited selection of industries, assumed to have many private workplaces, namely wholesale and retail, construction, manufacturing, commercial services and transport and storage. Results from these analyses are found in Supplementary Figure 4. We found a higher effect of IA within this subgroup, more akin to the effect seen in men in the main article.

S.6 Sensitivity analysis 5: Analysis with initial SA spells related to pregnancy, childbearing and family planning removed

Since our study population were in an age span 28-42, a high proportion of the initial SA cases were related to pregnancy, childbearing and family planning. We removed 20355 cases with such diagnosis types and ran the analysis for the remaining women. The results can be seen in Supplementary Figure 5. As can be seen in the Figure, there were an increased effect of IA among the remaining women after these diagnoses were removed.

Roughly one month after inclusion, we found that by removing these cases, the effect of IA on work was increased in the range of 0.4-0,6 pp higher probability of work, while being offset by similar reduction for being in SA. The effect difference is noticeable smaller and almost non-existent 100 days into the study and this pattern is seen for a good hundred days going forward. Towards the end of the study, we again see some increase in the effect on work in this limited stratum, but now the increased effect on work is offset by an even greater reduction in non-employment than seen in the full female population.

S.7 Sequence analysis of state trajectories within different industries

To illustrate how typical patterns of individual state trajectories differ between different industries and IA status, we made so-called sequence plots using the TraMineR package in R. Different industries with similar characteristics were grouped together. In order to plot individual trajectories with some form of clarity, we based the plots on 500 randomly sampled individuals within each stratum. The sequence plots are displayed in Supplementary Figure 6. Here, the state histories are sorted backwards after similar states, and indications of some differences in common patterns between the industries can be seen.

In all industries, the by far most common type of individual state history is that consisting of a shorter period of full-time SA, typically less than 2 months, followed by an uninterrupted stay in the work state. Furthermore, the increased amount of work seen in the IA intervention group, seems to come from individuals who are not in work at the final observation day, but rather have varying length of work periods in the middle of the observation time span. Partial SA appears to be less frequently used within construction and manufacturing industries.

S.8 Barplot showing number of individuals in the six biggest industries

The barplot in Supplementary Figure 7 illustrates the segregated labour market, where women typically work within health and education, while men are overrepresented in construction, manufacturing and transport industries.

References

[1] Hougaard P. Multi-state models: a review. Lifetime Data Analysis. 1999;5(3):239–264.

[2] Andersen PK, Keiding N. Multi-state models for event history analysis. Stat Methods Med Res. 2002;11(2):91–115.

[3] Putter H, Fiocco M, Geskus RB. Tutorial in biostatistics: competing risks and multi-state models. Stat Med. 2007;26(11):2389–2430.

[4] Aalen OO, Borgan Ø, Gjessing H. Survival and event history analysis: a process point of view. New York, NY: Springer; 2008.

[5] Hernan M, Robins JM. Causal Inference. Boca Raton: Chapman & Hall/CRC; 2020.

[6] Lipsitch M, Tchetgen ET, Cohen T. Negative controls: a tool for detecting confounding and bias in observational studies. Epidemiology (Cambridge, Mass). 2010;21(3):383.

[7] Gabadinho, A., G. Ritschard, N.S. Müller and M. Studer. Analyzing and Visualizing State Sequences in R with TraMineR. Journal of Statistical Software. 2011; 40(4).

Tables

Supplementary Table 1 Total number of transitions made by the study population of n = 187 930 individuals, over 90 231 405 person days, during individual follow-up of up to 500 days after entering full-time sickness absence (SA).

**FROM \ TO**	Full-time SA	Work	Graded SA	Non-employment	Education	Death
Full-time SA	0	197 774	32939	18760	492	163
Work	64 888	0	18562	37074	2003	78
Graded SA	9127	35254	0	1853	49	0
Non-employment	5902	28468	750	0	1015	34
Education	131	1526	31	737	0	0
Death	0	0	0	0	0	0

Figures

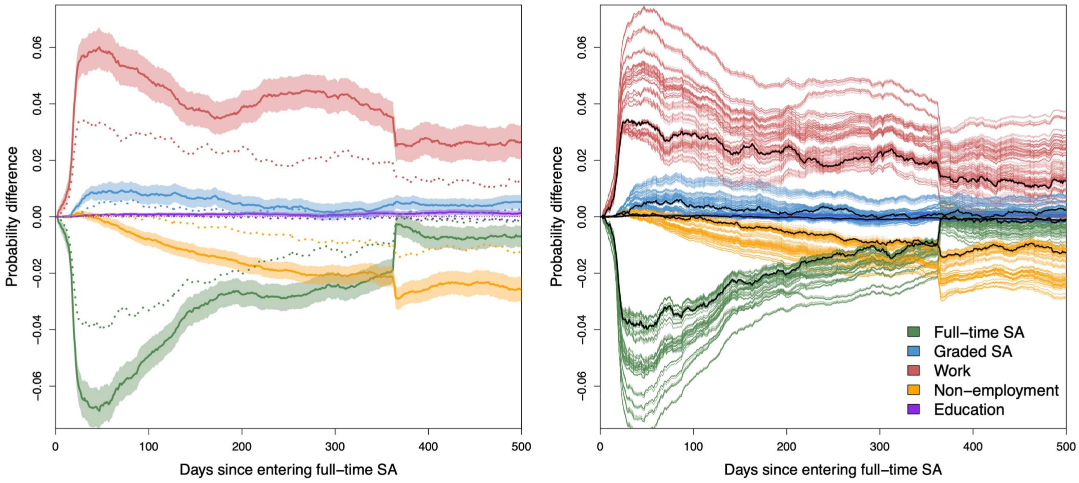

Supplementary Figure 1: Differences in estimated state probabilities, $\hat{\boldsymbol{\pi}}^1(t) - \hat{\boldsymbol{\pi}}^0(t)$, for the full study population, after entering long-term full-time sickness absence (SA), where superscript 1 denotes having the Inclusive Working Life Agreement (IA) and superscript 0 denotes not having IA. Faded areas are 95 % confidence intervals based on 1000 bootstrap samples. **Left:** The full-drawn lines are unadjusted estimates, while the dotted lines show the inverse probability of treatment (IPT) weighted estimates from the main article. **Right:** The black lines illustrate the IPT weighted estimates from the primary analysis in the main paper and colored lines are IPT weighted estimates from using different subsets of the main adjustment set when constructing the weights.

Hoff R, *et al. BMJ Open* 2022; 12:e062558. doi: 10.1136/bmjopen-2022-062558

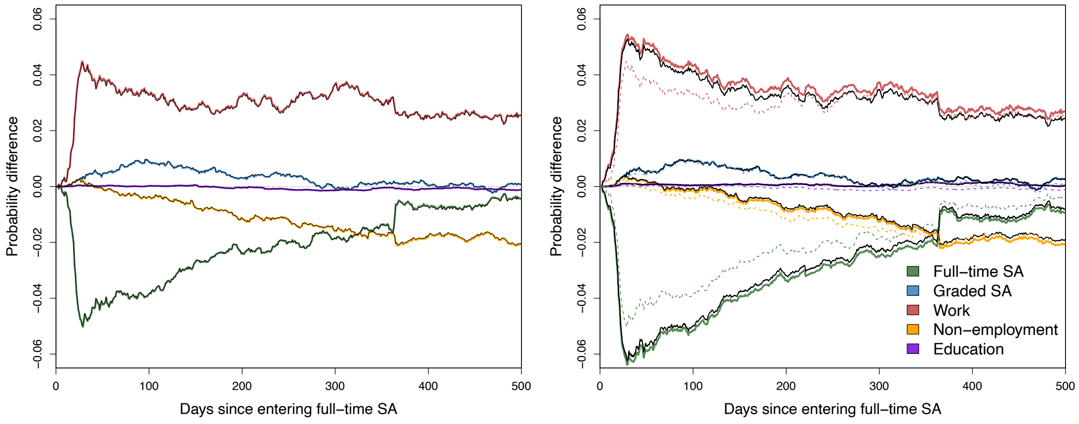

Supplementary Figure 2: Difference in estimated state probabilities, $\hat{\boldsymbol{\pi}}^1$ (t) − $\hat{\boldsymbol{\pi}}^0$ (t), for men who completed military conscript examinations, after entering long-term full-time sickness absence (SA), where superscript 1 denotes having the Inclusive Working Life Agreement (IA) and superscript 0 denotes not having IA. **Left:** The colored lines are estimates adjusted for confounding by inverse probability of treatment (IPT) weighting using the main confounder set. Black lines show the estimates from when conscript variables IQ score, BMI, physical stamina test and military eligibility test were also adjusted for. **Right:** The black lines show estimates where also conscript variables IQ score, BMI, physical stamina test and military eligibility test were adjusted for but leaving out the covariates for education and workplace industry. Full drawn colored lines are estimates adjusted for confounding by IPT weighting, leaving out education and workplace industry from the confounder set. Dotted colored lines are original estimates adjusted for confounding using the full main adjustment set.

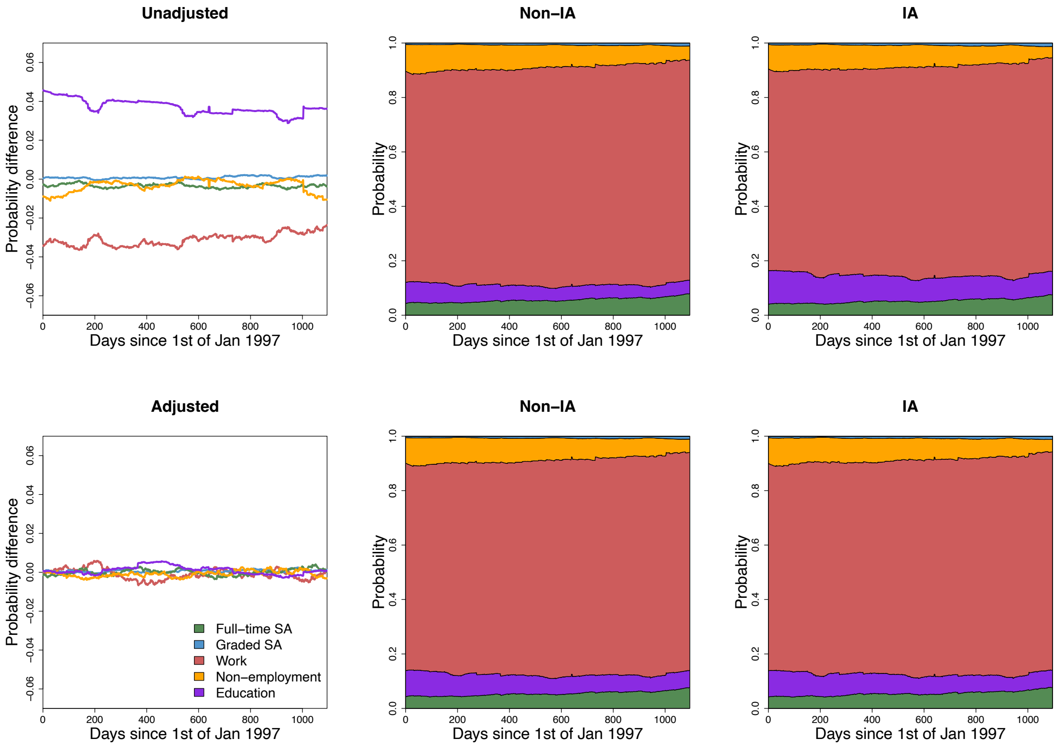

Supplementary Figure 3: The left column shows differences in state probabilities, $\hat{\boldsymbol{\pi}}^1(t) - \hat{\boldsymbol{\pi}}^0(t)$, where superscript 1 denotes having the Inclusive Working Life Agreement (IA) and superscript 0 denotes no IA. The middle and right column show state occupation probabilities for no IA and IA respectively. The top row shows unadjusted estimates, while the bottom row shows estimates adjusted for confounding using inverse probability of treatment (IPT) weighting.

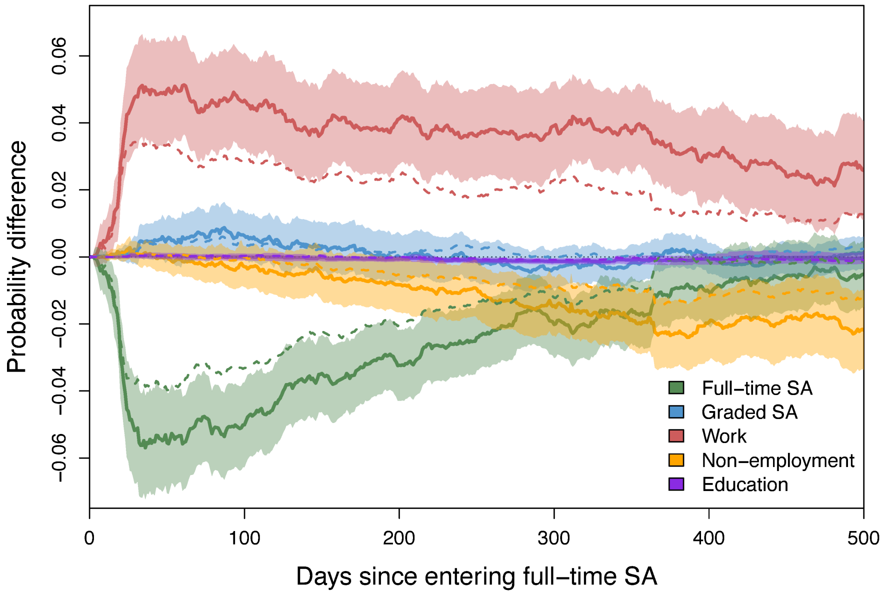

Supplementary Figure 4: Difference in estimated state probabilities, $\hat{\boldsymbol{\pi}}^1(t) - \hat{\boldsymbol{\pi}}^0(t)$, after entering long-term full-time sickness absence (SA), where superscript 1 denotes having the Inclusive Working Life Agreement (IA) and superscript 0 denotes not having IA. Estimates are adjusted for confounding using inverse probability of treatment (IPT) weighting. Faded areas are 95 % confidence intervals based on 1000 bootstrap samples. Full-drawn lines are estimates for the limited selection of industries, wholesale and retail, construction, manufacturing, commercial services and transport and storage, while the stippled lines show the estimates from the primary analysis (all industries).

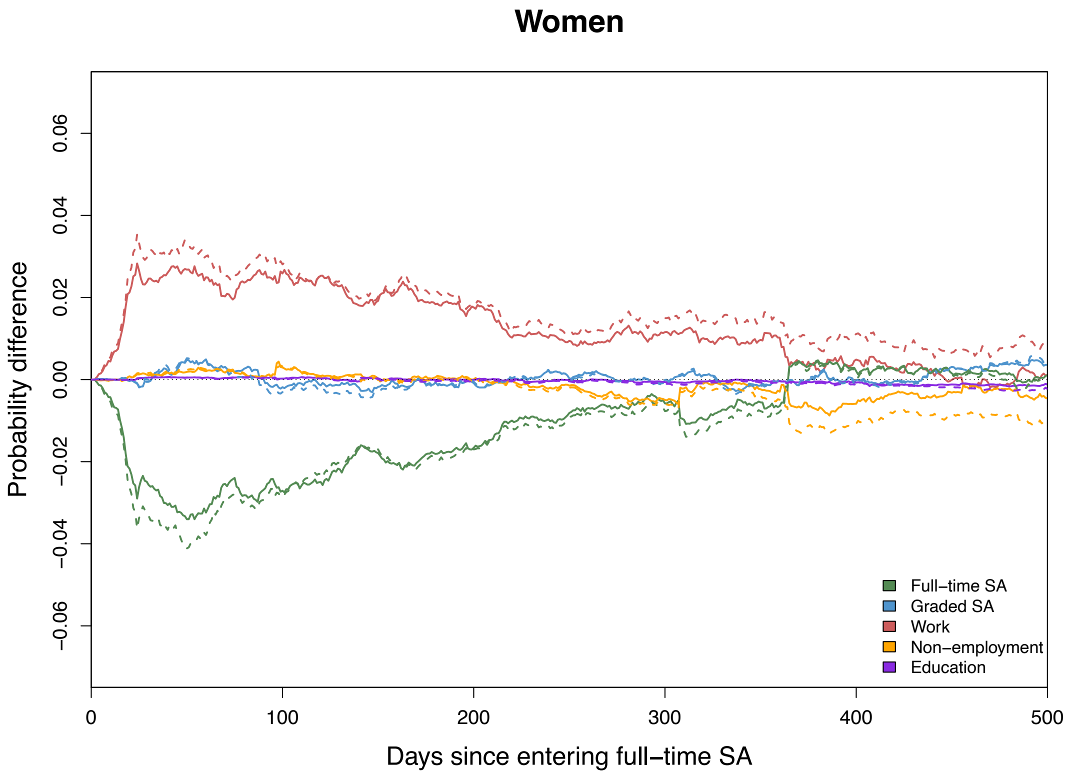

Supplementary Figure 5: Difference in estimated state probabilities, $\hat{\boldsymbol{\pi}}^1(t) - \hat{\boldsymbol{\pi}}^0(t)$, after entering long-term full-time sickness absence (SA), where superscript 1 denotes having the Inclusive Working Life Agreement (IA) and superscript 0 denotes not having IA. Estimates are adjusted for confounding using inverse probability of treatment (IPT) weighting. Faded areas are 95 % confidence intervals based on 1000 bootstrap samples. Full-drawn lines are estimates for all the women in the study population, while the stippled lines show updated estimates after removing the 20,355 women with initial SA diagnosis related to pregnancy, childbearing and family planning.

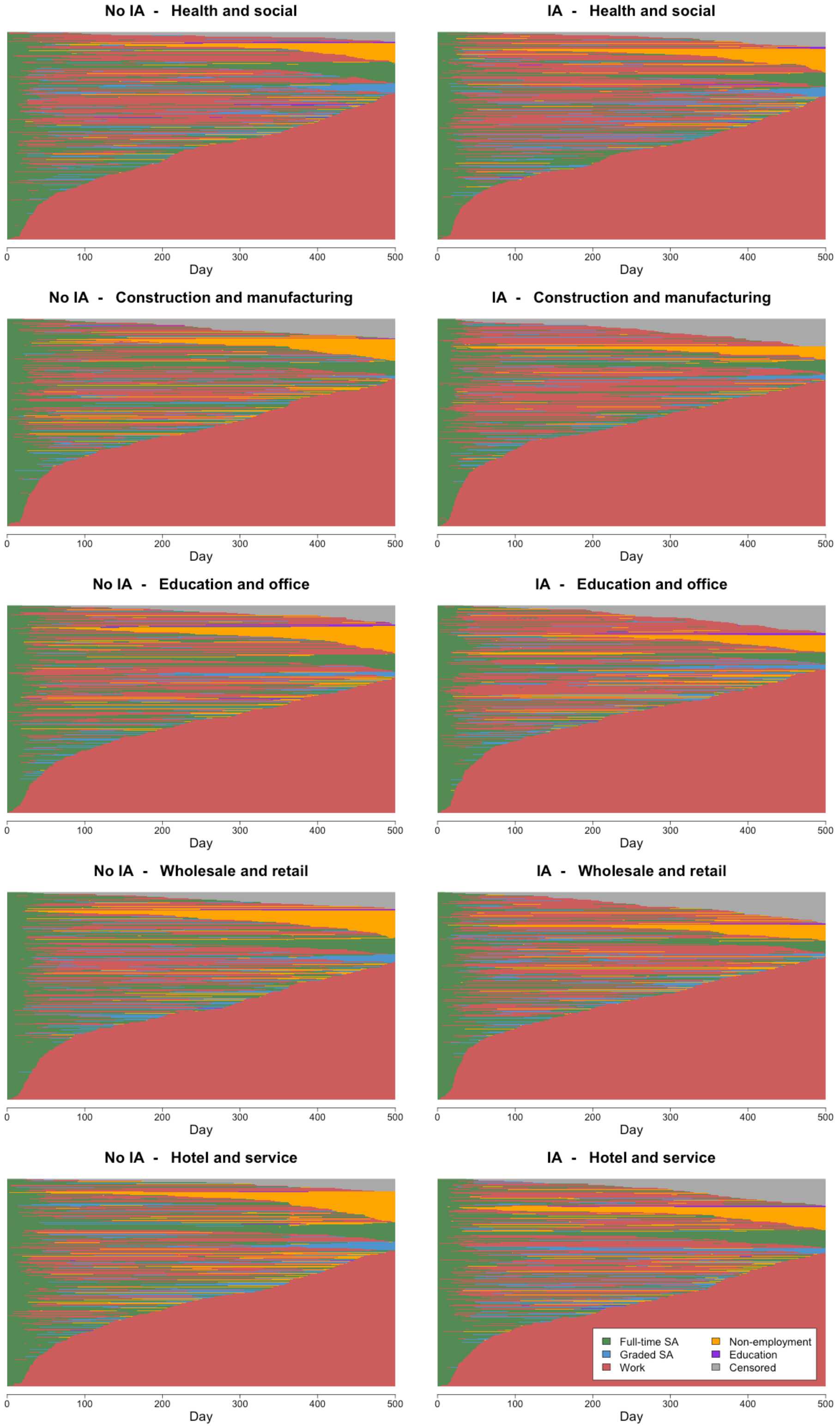

Supplementary Figure 6: State histories for 500 randomly sampled individuals within each group of industry. The sequences are ordered bottom up based the state occupied on the last observation day (day 500) and backwards on this state order: work, partial sick-leave, full-time sick-leave, non-employment, death, education and censoring.

Hoff R, *et al. BMJ Open* 2022; 12:e062558. doi: 10.1136/bmjopen-2022-062558

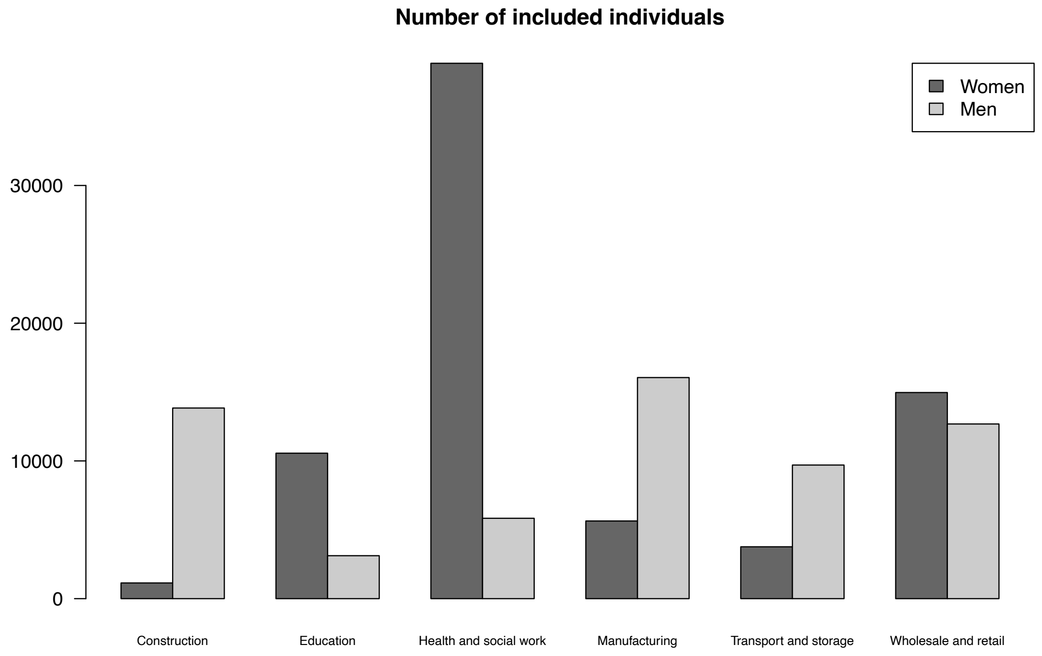

Supplementary Figure 7: Number of included individuals working in the six most represented industries of the study population.

Hoff R, *et al. BMJ Open* 2022; 12:e062558. doi: 10.1136/bmjopen-2022-062558
