# Supplementary material for: An initiative for a more inclusive working life and its effect on return-to-work after sickness absence: a multistate longitudinal cohort study
Source: BMJ Open. 2022 Nov 22;12(11):e062558. doi: 10.1136/bmjopen-2022-062558 (PMC9685245; doi:10.1136/bmjopen-2022-062558)
Supplement: Author's manuscript [file bmjopen-2022-062558.draft_revisions.pdf]

BMJ Open

BMJ Open is committed to open peer review. As part of this commitment we make the peer review history of every article we publish publicly available.

When an article is published we post the peer reviewers' comments and the authors' responses online. We also post the versions of the paper that were used during peer review. These are the versions that the peer review comments apply to.

The versions of the paper that follow are the versions that were submitted during the peer review process. They are not the versions of record or the final published versions. They should not be cited or distributed as the published version of this manuscript.

BMJ Open is an open access journal and the full, final, typeset and author-corrected version of record of the manuscript is available on our site with no access controls, subscription charges or pay-per-view fees (http://bmjopen.bmj.com).

If you have any questions on BMJ Open's open peer review process please email info.bmjopen@bmj.com

BMJ Open

An initiative for a more inclusive working life and its effect on return-to-work after sickness absence – a multi-state longitudinal cohort study

Journal:	*BMJ Open*
Manuscript ID	bmjopen-2022-062558
Article Type:	Original research
Date Submitted by the Author:	07-Mar-2022

Complete List of Authors:	Hoff, Rune; Oslo University Hospital<br>Maltzahn, Niklas; Oslo University Hospital; University of Oslo, Department of Biostatistics<br>Hasting, Rachel; STAMI<br>Merkus, Suzanne L; STAMI<br>Undem, Karina; STAMI<br>Kristensen, Petter; STAMI, Occupational Medicine and Epidemiology<br>Mehlum, Ingrid; STAMI, Occupational Medicine and Epidemiology; University of Oslo, Department of Community Medicine and Global Health<br>Gran, Jon; University of Oslo Faculty of Medicine, Department of biostatistics; Oslo University Hospital

Keywords:	OCCUPATIONAL & INDUSTRIAL MEDICINE, STATISTICS & RESEARCH METHODS, Health policy < HEALTH SERVICES ADMINISTRATION & MANAGEMENT

SCHOLARONE™ Manuscripts

I, the Submitting Author has the right to grant and does grant on behalf of all authors of the Work (as defined in the below author licence), an exclusive licence and/or a non-exclusive licence for contributions from authors who are: i) UK Crown employees; ii) where BMJ has agreed a CC-BY licence shall apply, and/or iii) in accordance with the terms applicable for US Federal Government officers or employees acting as part of their official duties; on a worldwide, perpetual, irrevocable, royalty-free basis to BMJ Publishing Group Ltd ("BMJ") its licensees and where the relevant Journal is co-owned by BMJ to the co-owners of the Journal, to publish the Work in this journal and any other BMJ products and to exploit all rights, as set out in our licence.

The Submitting Author accepts and understands that any supply made under these terms is made by BMJ to the Submitting Author unless you are acting as an employee on behalf of your employer or a postgraduate student of an affiliated institution which is paying any applicable article publishing charge ("APC") for Open Access articles. Where the Submitting Author wishes to make the Work available on an Open Access basis (and intends to pay the relevant APC), the terms of reuse of such Open Access shall be governed by a Creative Commons licence – details of these licences and which Creative Commons licence will apply to this Work are set out in our licence referred to above.

Other than as permitted in any relevant BMJ Author's Self Archiving Policies, I confirm this Work has not been accepted for publication elsewhere, is not being considered for publication elsewhere and does not duplicate material already published. I confirm all authors consent to publication of this Work and authorise the granting of this licence.

For peer review only

An initiative for a more inclusive working life and its effect on return-to-work after sickness absence – a multi-state longitudinal cohort study

Rune Hoff, PhD[1*], Niklas Maltzahn, MSc[1,2], Rachel Hasting, MSc[3], Suzanne L Merkus, PhD[3], Karina Undem, MSc[3], Petter Kristensen, PhD[3], Ingrid Sivesind Mehlum, PhD[3,4] and Jon Michael Gran, PhD[2,1]

[1*]Oslo Centre for Biostatistics and Epidemiology, Oslo University Hospital, Norway.
[2]Oslo Centre for Biostatistics and Epidemiology, Department of Biostatistics, Institute of Basic Medical Sciences, University of Oslo, Norway.
[3]Department of Occupational Medicine and Epidemiology, National Institute of Occupational Health, Oslo, Norway. [4]Department of Community Medicine and Global Health, Institute of Health and Society, University of Oslo, Norway.

*Corresponding author Rune Hoff. E-mail: rune.hoff@medisin.uio.no, Postal address: Oslo University Hospital, Sogn Arena, Oslo Centre for Biostatistics and Epidemiology, Pb 4950 Nydalen, 0424 Oslo, Norway.

Abstract

Objectives To reduce Sickness absence (SA) and increase work participation, the tripartite Agreement for a More Inclusive Working Life (IA) was established in Norway in 2001. IA companies have access to several measures to prevent and reduce SA, including special regulations and tailored support for employees with health problems. Our aim in this paper was to estimate the average effect of having access to IA at the time of entering a first SA on later return-to-work and on time spent in other work-related states. A secondary objective was to study how effects varied between women and men, and individuals with SA due to either musculoskeletal or psychological diagnoses.

Design Population based observational multi-state longitudinal cohort study.

Participants Each individual born in Norway 1967-1976 who entered full-time SA during 2004-2011, with limited earlier SA, was included (n = 187 930).

Outcome measures Individual multi-state histories containing dated periods of work, graded SA, full-time SA, non-employment and education.

Methods Individual characteristics and detailed longitudinal records of SA, work and education were obtained from population-wide registries and analysed in a multi-state model with 500 days of follow-up. The effect IA was assessed by estimating differences in state probabilities over time, using inverse probability weighting.

Results IA increased the probability of work after SA, with largest difference between groups after 29 days (3.4 percentage points higher (95% confidence interval: 2.5 – 4.3)). Differences in one-year expected length of stay was 8.4 additional days (4.9 – 11.9) in work, 7.6 (4.8 – 10.3) fewer days in full-time SA and 1.6 (-0.2 – 3.4) fewer days in non-employment. Similar trends were found within subgroups by sex, musculoskeletal and psychological diagnoses. The robustness of the findings was studied in sensitivity analyses.

Conclusion Measures to prevent and reduce SA, as given through IA, were found to improve individuals' return-to-work after entering SA.

Keywords: Workplace initiative, occupational health, working life policy

Word count: 3679

For peer review only - http://bmjopen.bmj.com/site/about/guidelines.xhtml

Strengths and limitations of this study

- This is the first study on the effects of the Norwegian population wide initiative to increase work-participation that analyses a large dataset of longitudinal multi-state outcomes.
- The data exhibit a high level of completeness and include also detailed covariate information.
- A limitation of the study is that we only have access to observational data and that the exposure was not randomised.

Introduction

Sickness absence (SA) from work is an important long-term health outcome for the individual, as well as a social and financial burden for society. [1– 3] It has been shown that while in SA, individuals are at greater risk of a more permanent exit from working life. [4, 5] SA increased greatly in Norway between 1995 and 2000, mainly due to an increase in long-term SA (more than 16 calendar days). The yearly average days of SA per worker in private workplaces, increased from 8.8 days to 12.9 days in the same period.[6] It should be noted that this increase came after a period of reduction in SA,[7] and that around one third of the increase can be explained by changes in the size and age of the workforce.[6]

In 2001, the Norwegian government reached out to major partners in working life, representing both workers and employers, with the intention of reducing SA and prolonging working life. This resulted in the tripartite Agreement for a More Inclusive Working Life (IA), which is still active, and included three operative goals: i) reduce SA by at least 20 %, ii) increase employment for individuals with functional limitations and iii) increase the average retirement age.[6] By 2004, 55% of workers were in IA companies.[8]

IA companies and their employees have access to several measures to reduce and prevent SA. These include special regulations and support from work centers that were established regionally by the Norwegian Welfare and Labour Administration (NAV). The support from these centers can be comprehensive, including tailored support for employees with health problems [9]. The measures include the possibility for employees in SA to use so-called active sickness benefits, where they may return to work and perform modified tasks while still receiving payments from NAV. Employees in IA companies are also allowed to have longer self-certified SA (up to eight calendar days, instead of three). The IA companies may receive refunds for measures related to aiding employees in returning to work. If it is impossible for an employee to return to the same position they had before SA, the IA companies have agreed to cooperate with NAV in assisting in retraining so that the employee can continue to work in the same company.

Since implemented, IA has not met the goals regarding SA, and systematic evaluations, although sparse, suggest limited or no effect on the risk of experiencing SA. [8, 10, 11] Studies of other general workplace interventions have found that they were generally not effective in reducing SA.[12] However, there are studies suggesting certain effects of workplace interventions on return-to-work. [13, 15]

The main objective of this study was to estimate the average effect of having access to IA at the time of entering a first SA on later return-to-work and on time spent in other work-related states for the following 500 days. To analyze such individual outcome trajectories in more detail than have been done before, we utilised a multi-state model,[14] capturing individuals' movement between the states of full-time SA, graded SA, work, non-employment and education over time. Inverse probability of treatment weighting was used to adjust for confounding between IA and multi-state outcomes.[15] The effect of IA has not previously been studied in detail at a population level using such methods. A secondary objective was to study how effects varied between women and men, and individuals with SA due to either musculoskeletal or psychological diagnoses.

Methods

Design, participants and exposure

To meet the study objectives, we utilized observational longitudinal registry data from a cohort consisting of all individuals born in Norway between 1967 and 1976 (626 928 individuals), as registered by the Medical Birth Registry of Norway. The same cohort is described in greater detail by Kristensen and Bjerkdal.[16] Given limited earlier SA (no SA in 2003), individuals were included in the analysis if they entered full-time SA (>16

For peer review only - http://bmjopen.bmj.com/site/about/guidelines.xhtml

days, counted from the first day), between January 1, 2004, when IA was properly implemented, and December 31, 2010. In total this comprised 187 930 persons. Baselines were set to the dates of the initial SA episode for all Individuals, who simultaneously had their IA exposure (yes/no) recorded, based on the IA status of the company they worked for, and were followed for 500 days or until death or administrative censoring on January 1, 2011. The choice of 500 days was a pragmatic choice, meant to cover the first full year and a short period beyond. The immediate period after the first year is of interest, as welfare policies impose a change in benefits received after a full year of receiving sick leave benefits. Moreover, for even longer follow-up periods, individuals' IA status would be more likely to change, making results from an analysis based on baseline IA status more difficult to interpret.

Patient and public involvement

Patients or the public were not involved in the design, or conduct, or reporting, or dissemination plans of our research.

Outcomes and confounders

The individual multi-state outcomes included dated periods of work, graded SA, full-time SA, non-employment, education and death, as shown in Figure 1. For this study, all possible trajectories from SA to other states and possibly back to SA again within the 500-day study period were considered. Individuals started in full-time SA and could move between states at any time.

Confounders we adjusted for were age, sex, education level, calendar year, company size, geographical region and type of industry, recorded at individuals' baseline. Education level was the individual's highest attained degree, separated into four categories: lower secondary, upper secondary, college and university. Type of industry covered 20 categories in the Statistical Classification of Economic Activities in the European Community.[17] Company size was based on yearly records of number of employees and company region covered middle, eastern, northern, southern and western parts of Norway.

Data sources and data preparation

Individual and company characteristics, IA status and longitudinal outcomes were available from Statistics Norway's event database of employment and welfare (FD-Trygd), The National Education Database and the registries of the Norwegian Labour and Welfare Administration. The data contained dated individual records of sick leave (>16 days) pension, work assessment allowance, disability pension, paid employment, and admittance to education programs. From these records, we generated individual longitudinal outcome histories of full-time SA, graded SA, work, education and non-employment. For this study, SA included periods eligible for either of three different types of pensions in the Norwegian welfare system: sick leave pension, work assessment allowance and disability pension, all attainable as full-time (100%) or graded (< 100%). Work referred to paid employment and included holidays and parental leave, and education covered admittance to officially licensed education programs. Periods without SA, work or education were considered as non-employment – for instance unemployment (job seekers) and those outside the labour market. Whenever different types of records occurred simultaneously on the same dates, records had precedence in the following order, from most to least important: SA, work and lastly education.

Subgroup and sensitivity analyses

In addition to the main analysis, separate analyses were done for women and for men, and for diagnosis group associated with initial SA (musculoskeletal or psychological). Musculoskeletal diagnoses consisted of ICPC-2 (International Classification of Primary Care) [18] codes starting with L, while psychological diagnoses referred to codes starting with P.

We conducted four sensitivity analyses. First, we compared results adjusting for subsets of the confounders, to see how omitted variables could bias results. In the second, we adjusted for additional potential confounders available for a male subgroup of the study population, describing IQ, physical stamina, BMI and eligibility for military. In the third, we used pre-baseline longitudinal outcomes between 1997-1999, as negative outcome controls.[19] If confounding was sufficiently adjusted for, state transition histories before baseline should be similar across IA groups after weighting with the same IPT weights as in the main analysis. In the fourth, we

For peer review only - http://bmjopen.bmj.com/site/about/guidelines.xhtml

analysed a subset of industries with mostly private companies. Closer descriptions of these sensitivity analyses are included in the supplementary material.

Estimands and statistical methods

Individual longitudinal outcomes were studied in hazard based multi-state models for time-to-event data.[14] By first fitting hazard models for each transition and then estimating state probabilities for all states in Figure 1. By state probabilities we here mean the probability of being in a particular state at a given time-point, which can be seen as an extension of regular survival probabilities (e.g., Kaplan-Meier curves). To assess the effect of IA, we sought to identify the average treatment effect, as the difference in state probabilities $\theta(t) = \pi^1(t) - \pi^0(t)$, where $\pi^a(t)$ are the marginal, overall, state probabilities if everyone in the population in question was fixed to IA status a (taking the values 1 and 0 for IA and no IA respectively). From the state probabilities we calculated the expected length of stay (ELOS) [20] in the different states over the first year, for the situations where everyone was fixed to a = 1 and a = 0. For a more formal specification of the target estimands and details on the statistical methods, see section S.1 in the supplementary material.

Adjusting for confounders was done by calculating baseline stabilized IPT weights,[21] also known as propensity score weights, and then fitting weighted additive hazards regression [22] models for every possible transition in Figure 1, adjusting only for IA status. To calculate the IPT weights we modelled IA status as a function of the baseline confounders in a logistic regression model. Since IA is an intervention at company level, we calculated 95% confidence intervals using a clustered bootstrap, resampling on companies with replacement.[23] All analyses were performed in R, on the TSD (Service for Sensitive Data) facilities, owned by the University of Oslo. R code is available on GitHub (see Declarations).

Results

Descriptive statistics

Over a total follow-up of 90 231 405 person days, the 187 930 individuals included made a total of 457 610 transitions, which are summarized in detail in Supplementary Table 1. A summary of the individual characteristics can be found in Table 1.

Table 1 Descriptive statistics for individuals working in companies with the Inclusive working life Agreement (IA), or without at the time of inclusion. The table also includes the inverse probability of treatment (IPT) weighted population.

	No IA, n (%)	IA, n (%)	No IA, n (%)	IA, n (%)
	n = 119 363	n = 68 567	*IPT Weighted*	*IPT Weighted*
**Sex**				
Women	57002 (0.48)	45139 (0.66)	63383 (0.53)	37907 (0.55)
Men	62361 (0.52)	23428 (0.34)	55037 (0.46)	30885 (0.45)
**Age at inclusion**				
28-32	33876 (0.28)	16336 (0.24)	31722 (0.27)	18619 (0.27)
33-37	60561 (0.51)	34485 (0.50)	60150 (0.50)	35121 (0.51)
38-43	24926 (0.21)	17746 (0.26)	26547 (0.22)	15052 (0.22)
**Inclusion year**				
2004	31255 (0.26)	12762 (0.19)	28556 (0.24)	17376 (0.25)
2005	25935 (0.22)	12671 (0.18)	24741 (0.21)	14278 (0.21)
2006	19812 (0.17)	11373 (0.17)	19757 (0.17)	11169 (0.16)
2007	14951 (0.13)	9895 (0.14)	15307 (0.13)	8769 (0.13)
2008	11955 (0.10)	9094 (0.13)	12704 (0.11)	7258 (0.11)
2009	10082 (0.08)	8029 (0.12)	11234 (0.09)	6366 (0.09)
2010	5373 (0.05)	4743 (0.07)	6121 (0.05)	3578 (0.05)
**Education**				
Lower secondary	39162 (0.33)	17005 (0.25)	35319 (0.30)	19753 (0.29)
Upper secondary	52977 (0.44)	24137 (0.35)	48767 (0.41)	28885 (0.42)
College	23393 (0.20)	23754 (0.35)	28851 (0.24)	17392 (0.25)
University	3831 (0.03)	3671 (0.05)	5483 (0.05)	2763 (0.04)
**Industry**				
Agriculture/forestry	1406 (0.01)	106 (0)	963 (0.01)	720 (0.01)

For peer review only - http://bmjopen.bmj.com/site/about/guidelines.xhtml

Commercial services	7015 (0.06)	1811 (0.03)	5633 (0.05)	3381 (0.05)
Construction	12357 (0.10)	2763 (0.04)	9571 (0.08)	4785 (0.07)
Education	3959 (0.03)	9624 (0.14)	9594 (0.08)	4941 (0.07)
Scientific service work	5475 (0.05)	853 (0.01)	4006 (0.03)	2120 (0.03)
Electricity supply	387 (0)	317 (0)	473 (0)	289 (0)
Entertainment	1502 (0.01)	379 (0.01)	1203 (0.01)	944 (0.01)
Financial	1701 (0.01)	1189 (0.02)	1923 (0.02)	1282 (0.02)
Health and social work	16658 (0.14)	27779 (0.41)	25274 (0.21)	15954 (0.23)
Hotel and restaurant	3873 (0.03)	856 (0.01)	2994 (0.03)	1543 (0.02)
Information/communication	5087 (0.04)	1432 (0.02)	4126 (0.03)	2228 (0.03)
Manufacturing	14306 (0.12)	7560 (0.11)	13694 (0.11)	7429 (0.11)
Mining/quarrying	2584 (0.02)	1075 (0.02)	2331 (0.02)	1394 (0.02)
Other service	2166 (0.02)	554 (0.01)	1731 (0.01)	1007 (0.01)
Public administration	3765 (0.03)	5528 (0.08)	6850 (0.06)	3756 (0.05)
Real estate	1116 (0.01)	90 (0)	768 (0.01)	493 (0.01)
Transport and storage	10150 (0.09)	3292 (0.05)	8687 (0.07)	4952 (0.07)
Unknown or private households	91 (0)	5 (0)	61 (0)	50 (0)
Water supply	982 (0.01)	222 (0)	772 (0.01)	560 (0.01)
Wholesale and retail	24783 (0.21)	3132 (0.05)	17763 (0.15)	10964 (0.16)
**Company size**				
1 – 9	31575 (0.26)	3233 (0.05)	22050 (0.18)	12281 (0.18)
10 – 49	48126 (0.40)	20514 (0.30)	43030 (0.36)	24979 (0.36)
50 – 249	12555 (0.11)	19437 (0.28)	20198 (0.17)	11599 (0.17)
≥ 250	27107 (0.23)	25383 (0.37)	33142 (0.28)	19934 (0.29)
**Region**				
East	55898 (0.47)	29625 (0.43)	55702 (0.47)	31407 (0.46)
Middle	10695 (0.09)	6577 (0.10)	10405 (0.09)	6180 (0.09)
North	14471 (0.12)	7700 (0.11)	14002 (0.12)	8849 (0.13)
South	9459 (0.08)	6399 (0.09)	9773 (0.08)	5692 (0.08)
West	28840 (0.24)	18266 (0.27)	28538 (0.24)	16665 (0.24)
**Diagnosis**				
Musculoskeletal	43419 (0.36)	21570 (0.31)	40752 (0.34)	24199 (0.35)
Other types	53417 (0.45)	34490 (0.50)	55200 (0.46)	32385 (0.47)
Psychological	22527 (0.19)	12507 (0.18)	22467 (0.19)	12209 (0.18)

First, we fitted a multi-state model without covariates, to calculate state probabilities in the entire cohort, regardless of IA status. A stacked probability plot is shown in Figure 2. All individuals started in full-time SA and could thereafter move to other states. The proportion of individuals who had returned to work reached 50.6 % (95% confidence interval (CI): 50.4 – 50.8) after 50 days, and 64.7 % (64.5 – 64.9) after 100 days. Just before passing one year, 77.0 % (76.8 – 77.2) were in work, 3.3 % (3.2 – 3.4) were in graded SA and 9.9 % (9.8 – 10.0) in full-time SA. At the same time, 9.2 % (9.0 – 9.3) was in non-employment and in 0.5 % (0.47 – 0.53) in education. After one year we see distinct shifts in the probabilities, coinciding with maximum allowed days of consecutive sick leave pension. After this, individuals either return to work or typically receive other welfare benefits, including work assessment allowance.

Main analysis

To assess the effect if IA versus no IA we compared state probabilities in an IPT weighted multi-state model. A summary of individual characteristics in the weighted population can be found in Table 1. Figure 3 shows the absolute difference in state probabilities for IA versus no IA over the follow-up period, with 95% CI based on 1000 bootstrap samples. In the figures, death was left out, as differences here were negligible. The figure shows how IA increased the probability of return-to-work and decreased the probability of full-time SA in the period following an initial SA episode. Peak effect of IA on return-to-work occurred after one month, where the probability was about 3.4 percentage points (pp) higher with IA.

The difference declined to below 2 pp after one year. The effect on full-time SA followed an opposite pattern for the first 2- 3 months and declined to zero after one year. The effect on graded SA was small (increase), but the confidence intervals included zero. The effect on non-employment was small initially but amounted to around 1 pp reduction after one year.

The average individual effects of IA versus no IA are summarized in terms of ELOS in Table 2. Here, we see that the estimated difference between IA and no IA for first year ELOS in work was 8.4 more days (95% CI: 4.9

For peer review only - http://bmjopen.bmj.com/site/about/guidelines.xhtml

– 11.9 days), the difference was 7.6 (4.8 – 10.3) fewer days in full-time SA and 1.6 (-0.2 – 3.4) fewer days in non-employment. Differences for graded SA and education were smaller.

Table 2 Expected length of stay (ELOS) in days, for one year after entering long-term full-time sickness absence (SA) from work with 95% confidence intervals based on 1000 bootstrap samples.

	**IA**	**No IA**	**Difference**
**Full study population**			
Full-time SA	88.8 (87.2 – 90.5)	96.4 (95.3 – 97.5)	-7.6 (-10.3 – -4.8)
Graded SA	18.1 (17.2 – 19)	17.3 (16.7 – 17.9)	0.8 (-0.7 – 2.2)
Work	240.8 (238.7 – 242.8)	232.4 (230.9 – 233.8)	8.4 (4.9 – 11.9)
Non-employment	16.2 (15.1 – 17.3)	17.8 (17.1 – 18.5)	-1.6 (-3.4 – 0.2)
Education	0.9 (0.7 – 1.1)	1 (0.8 – 1.1)	-0.1 (-0.5 – 0.3)
**Women**			
Full-time SA	94.2 (92.3 – 96.1)	100 (98.3 – 101.7)	-5.8 (-9.4 – -2.2)
Graded SA	21.9 (20.7 – 23.2)	21.9 (21.1 – 22.8)	0 (-2.1 – 2.1)
Work	232.3 (230 – 234.6)	226.3 (224.2 – 228.4)	6 (1.6 – 10.4)
Non-employment	15.3 (14 – 16.6)	15.5 (14.4 – 16.5)	-0.2 (-2.5 – 2.1)
Education	1.2 (0.8 – 1.6)	1.2 (1 – 1.5)	0 (-0.6 – 0.6)
**Men**			
Full-time SA	82.9 (80.3 – 85.5)	92.3 (91 – 93.6)	-9.4 (-13.3 – -5.5)
Graded SA	13.4 (12.2 – 14.7)	12 (11.4 – 12.6)	1.4 (-0.4 – 3.2)
Work	250.3 (247.2 – 253.5)	239.1 (237.4 – 240.8)	11.2 (6.4 – 16.1)
Non-employment	17.5 (15.6 – 19.3)	20.6 (19.8 – 21.4)	-3.1 (-5.8 – -0.5)
Education	0.6 (0.3 – 0.8)	0.7 (0.6 – 0.9)	-0.1 (-0.6 – 0.3)
**Musculoskeletal**			
Full-time SA	92 (89.2 – 94.8)	100.3 (98.4 – 102.1)	-8.3 (-12.9 – -3.7)
Graded SA	20.2 (18.7 – 21.8)	18.7 (17.9 – 19.6)	1.5 (-1 – 3.9)
Work	236 (232.4 – 239.6)	227.5 (225.2 – 229.8)	8.5 (2.6 – 14.4)
Non-employment	16.1 (14.3 – 17.8)	17.7 (16.7 – 18.6)	-1.6 (-4.3 – 1.1)
Education	0.7 (0.4 – 1.1)	0.8 (0.5 – 1)	0 (-0.6 – 0.5)
**Psychological**			
Full-time SA	104.3 (100.1 – 108.6)	115.4 (112.7 – 118.1)	-11.1 (-18 – -4.2)
Graded SA	22.4 (20.2 – 24.5)	21.2 (19.9 – 22.5)	1.2 (-2.2 – 4.6)
Work	213 (207.9 – 218.1)	198.9 (195.9 – 201.9)	14.1 (6 – 22.3)
Non-employment	23.9 (20.7 – 27.1)	27.7 (26 – 29.3)	-3.7 (-8.6 – 1.2)
Education	1.1 (0.6 – 1.7)	1.7 (1.3 – 2.2)	-0.6 (-1.6 – 0.4)

Subgroup analyses

Results from the subgroup analyses are plotted in Figure 3. Similar effect patterns as before were found, however, effects of IA were higher for men than for women. Effects were also higher in both selected diagnosis groups compared to in the overall analysis, although only slightly for musculoskeletal diagnoses.

For women, the effect of IA on return-to-work from full-time SA, reached a peak after 25 days, where the probability was 2.8 pp (95%CI: 1.9 – 3.7) higher with IA. The effect remained close to 2 pp increase for roughly 200 days, before declining to around 1 pp one year after inclusion. After one year, the effect was close to zero. The effect on work was almost entirely offset by reduced probability of full-time SA, while effects on other

For peer review only - http://bmjopen.bmj.com/site/about/guidelines.xhtml

states were smaller. The peak effect for men on return-to-work were 29 days after starting full-time SA, with 4.5 pp (3.0 – 6.0) higher probability for men with IA. Contrary to in women, the effect on work stayed positive, never dropping below 2 pp, the entire period. The effect on work was mostly offset by reduced probability of full-time SA, but with more noticeable effects on other states compared to women. There was a small increase in graded SA (after initially starting in full-time). Furthermore, there was a clear reduction in non-employment of about 2 pp after one year for men from having IA at the time of the initial SA.

Workers with musculoskeletal illnesses as cause for their initial SA, reached peak effect of IA on return-to-work after 27 days, where the probability was 3.7 pp (2.4 – 5.0) higher than without IA. This effect magnitude was relatively stable until around day 100, from where it declined and then fluctuated around 2 pp to the end of follow-up. The effect on work was mostly offset by reduced probability of SA, though a slight increase for graded SA was seen and a 2 pp lower probability of non-employment after one year.

In the group with psychological diagnoses, the effect on return-to-work reached a maximum after 37 days, with 5.6 pp (3.5 – 7.7) higher probability with IA. Compared to other subgroups, effects varied much more over time. From the maximum effect of 5.6 pp, it dropped down to a 3 pp increase after one month, before going back to over 5 pp three months later. The effect then varied between 3 and 5 pp before dropping down to 2 pp after one year. In the first year, the effect on return-to-work was mostly offset by reduced full-time SA, but a small increase in graded SA and reduction in non-employment was seen. One year after an initial SA episode due to psychological illnesses, the probability of non-employment was more than 2 pp lower with IA.

Average effects of IA versus no IA for subgroups are summarized in Table 2 in terms of ELOS over the first year. For women, we found that IA led to on average 5.8 (95% CI: 2.2 – 9.4) fewer days in full-time SA and 6 (1.6 – 10.4) more days in work. ELOS in other states were not affected. In men, effects were bigger. Men with IA could expect 9.4 (5.5 – 13.3) fewer days in full-time SA and 11.2 (6.4 – 16.1) more days in work. There was a small increase in ELOS for graded SA of 1.4 days (-0.4 – 3.2), but the confidence interval included zero. Non-employment was lowered by 3.1 days (-5.8 – -0.5). For individuals with initial SA due to musculoskeletal illnesses, IA led to on average 8.3 (3.7 – 12.9) fewer days in full-time SA and 8.9 (3.3 – 14.5) more days in work. ELOS differences for graded SA and non-employment were small and both confidence intervals included zero. Individuals with psychological diagnoses had the biggest differences in ELOS. IA led to on average 14.1 (6 – 22.3) more days in work over the first year, and 11.1 (4.2 – 18) fewer days of full-time SA. Non-employment was reduced by 3.7 days for the psychological diagnosis group, but the confidence interval included zero.

Sensitivity analyses

To study the impact of covariate adjustment and to investigate robustness of our results, we conducted a series of sensitivity analyses, as described earlier. The analyses all supported our main findings. Detailed results from these analyses are described in section S.2 to S.5 in the supplementary material and shown in Supplementary Figure 1-4.

Discussion

We found that having access to the Norwegian IA Agreement, with the within measures for preventing and reducing SA at the time of initiating a first long- term SA episode, had a positive effect on the probability of later work and decreased the probability of later full-time SA during the first 500 days after first entering SA. The probability of later non-employment was also reduced, particularly after 1 year. Larger effects were found for men than for women, and among individuals with musculoskeletal or psychological diagnoses at initial SA. For women, the effect of IA was negligible after one year, while for men, the effect remained also after the first year.

A reduction in full-time SA and a slight increase in graded SA follow the first IA goal of reducing SA. Additionally, it is worth noticing that non-employment was reduced with IA, especially for men, one year after entering SA. From a health perspective, the underlying intention of IA is to reduce SA by remaining in work, also after the first year when the right to certain SA benefits in Norway ceases. Our results show that having IA contributes to reducing SA by keeping people in work, rather than by exiting work. Furthermore, while previous studies found that IA enterprises may have higher SA, or that IA has no effect on the occurrence of SA, [8, 11] our study shows that once on SA, IA contributed towards reducing the number of SA days. This could indicate that IA had a larger effect on SA duration than on the occurrence of SA.

For peer review only - http://bmjopen.bmj.com/site/about/guidelines.xhtml

We estimated effects of having access to IA and did not investigate which measures within IA that had most effect or whether workers had access to them. There is however supporting evidence that workplace health-promotion interventions may prolong working life in some labour market segments.[24] Also, as IA has been gradually introduced at a national level in Norway over the study period and beyond, it is likely that IA has had an impact on SA and return-to-work at a societal level. This effect is not picked up in our study, as we consider individual differences in having IA or not over the study period.

A strength of this study is the use of a large cohort with high completeness, with detailed information on individual longitudinal work-participation and a large set of confounders. Another strength is the use of multi-state modelling. We believe that the study serves as a good example of how longitudinal data from multiple registries can be used to construct detailed outcome trajectories and that IPT weighted multi-state models offer a suitable way of estimating effects on such outcomes. The approach allows us to analyse more detailed long-term work-related outcomes than traditional approaches, and to calculate various relevant outcome measures, ELOS being one example. There are also various limitations. Most importantly this is an observational study, and access to the measures given through IA was not randomized, but subject to the decision of the company signing up to IA. Even though adjustment for various assumed confounding variables was made, there is no guarantee that residual confounding is not present. However, sensitivity analyses were not able to detect residual confounding in settings where more detailed confounder information was available. The negative control by analysing pre-exposure multi-state histories indicated the same. Note that if the negative control had showed different pre-exposure histories, even after initial confounder adjustment, these histories could also be adjusted for as additional baseline information.

Given the assumptions of no unmeasured confounding, positivity and consistency, the IPT weighted multi-state model aim to mimic the scenario one would see if access to IA was randomized at baseline (time of first long-term SA episode). Note also that we implicitly assume that IA exposure acts as if given at first SA. As our analyses are restricted to individuals without any recent SA history, we believe this to be a reasonable assumption. The mechanisms through which IA potentially promotes faster return-to-work are many. One can assume that some would be connected to individual measures undertaken at IA workplaces, while others are related to measures available for IA workplaces in general. The difference in effect between genders was substantial, and the greater effect of IA found for men compared to women could imply that typical male dominated workplaces and jobs had more to gain from having IA with regards to return-to-work. Greater effects among individuals who had musculoskeletal or psychological diagnoses were as expected, as these are patient groups that IA measures were specifically aimed at.[6]

IA is specific for Norway, and it is not certain that similar effects would have been seen in other countries. Norway is known for having a generous welfare system and high protection for individual workers. Additionally, the cohort studied was young (28-43 years), and results do not necessarily generalise to older populations. Therefore, future work should focus on understanding how the effects of measures for preventing and reducing SA vary with different populations and worker characteristics.

Contributorship statement

All authors contributed to the conception of the study, interpretation of the data and the study design. Data preparation was performed by Rune Hoff and Niklas Maltzahn. All statistical analyses were performed by Rune Hoff. The first draft of the manuscript was written by Rune Hoff. All authors commented on previous versions and contributed to revised versions. All authors read and approved the final manuscript.

Competing interests

None declared.

Funding

This work was supported by the Research Council of Norway (Grant number 273 674).

Data sharing statement

For peer review only - http://bmjopen.bmj.com/site/about/guidelines.xhtml

No data are available. The data underlying this article are considered sensitive and subject to strict regulation and cannot be shared publicly due to privacy of individuals included in the study.

R code for preparing individual multi-state histories and performing multi-state models is available on GitHub, see https://github.com/runhoff/IA/.

Ethics statements

Consent to participate Not applicable.

Consent for publication Not applicable.

Ethical approval Not applicable.

Acknowledgments

The late Professor Emeritus Tor Bjerkedal (1926–2015) conceived the idea for and took part in establishing this registry-based cohort in 2002.

For peer review only

References

[1] Henderson M, Glozier N, Elliott K H. Long term sickness absence. *BMJ* 2005;330:802–803.

[2] Waddell G, Burton A K. Is Work Good for Your Health and Well-being? The Stationery Office, London 2006.

[3] Gründemann R W M, van Vuuren C V. Preventing Absenteeism at the Workplace: A European Portfolio of Case Studies. European Foundation for the Improvement of Living and Working Conditions, Dublin 1998.

[4] Kivimäki M, Ferrie J E, Hagberg J, et al. Diagnosis-specific sick leave as a risk marker for disability pension in a Swedish population. *BMJ* 2007;61(10):915–920.

[5] Karlsson N E, Carstensen J M, Gjesdal S, et al. Risk factors for disability pension in a population-based cohort of men and women on long-term sick leave in Sweden. *Eur. J. Public Health* 2008;18(3):224–231.

[6] Arbeids- og sosialdepartementet: Intensjonsavtale om et inkluderende arbeidsliv - budsjettmessige konsekvenser mv. (2001). https://www. regjeringen.no/no/dokumenter/stprp-nr-1-tillegg-nr-1-2001-2002-/ Accessed 2020-08-03.

[7] Gjesdal S. Sykefraværets utvikling i Norge 1975–2002. *Tidsskr. Nor. Lægeforen.* 2005;125:742–5.

[8] Lie A. ”Inclusive Working Life” in Norway” – Experience from ”Models of Good Practice” *Enterprises. Croat. Med. J.* 2008;49(4):553–560.

[9] Ministry of Labour. Letter of Intent regarding a more inclusive working life 2006–2009 [2007]. https://www.regjeringen.no/globalassets/ upload/aid/temadokumenter/velferd/ia/a more inclusive working life 2006-2009.pdf Accessed 2021-10-04.

[10] Foss L, Gravseth H M, Kristensen P, et al. “Inclusive working life in Norway”: a registry-based five-year follow-up study. *J. Occup. Med. Toxicol*. 2013;8(1):1–8.

[11] Hanvold T N, Kristensen P, Corbett, K, et al. Long-term sickness absence among young and middle-aged workers in Norway: the impact of a population-level intervention. *BMC Public Health* 2020:20(1);1–12.

For peer review only - http://bmjopen.bmj.com/site/about/guidelines.xhtml

[12] Odeen M, Magnussen L H, Mæland S, et al. Systematic review of active workplace interventions to reduce sickness absence. *Occup. Med*. 2013;63(1):7–16.

[13] Steenstra I A, Anema J R, Van Tulder M W, et al. Economic evaluation of a multi-stage return to work program for workers on sick-leave due to low back pain. *J. Occup. Rehabil*. 2006;16(4):557–578.

[14] Andersen P K, Keiding N. Multi-state models for event history analysis. *Stat Methods Med Res* 2002;11(2):91–115.

[15] Gran J M, Lie S A, Øyeflaten I, et al. Causal inference in multi-state models–sickness absence and work for 1145 participants after work rehabilitation. *BMC Public Health* 2015;15(1):1082.

[16] Kristensen P, Bjerkedal T. Educational attainment of 25 year old Norwegians according to birth order and gender. *Intelligence* 2010;38(1):123–136.

[17] Carré H. Statistical Classification of Economic Activities in the European Community. Publications Office of the European Union, Luxembourg 2008.

[18] Verbeke M, Schrans D, Deroose S, et al. The international classification of primary care (icpc-2): an essential tool in the EPR of the GP. *Stud. Health Technol. Inform*. 2006;124:809.

[19] Lipsitch M, Tchetgen E T, Cohen T. Negative controls: a tool for detecting confounding and bias in observational studies. *Epidemiology* 2010;21(3):383.

[20] Grand M K, Putter H. Regression models for expected length of stay. *Stat. Med.* 2016;35(7):1178–1192.

[21] Hernan M, Robins J M. Causal Inference: What If. Chapman & Hall/CRC, Boca Raton 2020.

[22] Aalen O O, Borgan Ø, Gjessing H. Survival and Event History Analysis: a Process Point of View. Springer, New York, NY 2008.

[23] Xiao Y, Abrahamowicz M. Bootstrap-based methods for estimating standard errors in Cox's regression analyses of clustered event times. *Stat. Med.* 2010;29(7-8):915–923.

[24] Nielsen R A, Midtsundstad T I. Do workplace health-promotion interventions targeting employees with poor health reduce sick-leave probability and disability rates? *Scand. J. Public Health* 2020;49(2):219–227

Figures

Figure 1 Multi-state model showing possible transitions, after starting in in full-time sickness absence (SA), between full-time SA, graded SA, work, non-employment and education. Note that death was a competing transition in all states, but left out of the figure

Figure 2 Stacked probability plot of estimated unadjusted state probabilities, for the full study population (both intervention groups, n = 187 930), after entering full-time sickness absence (SA).

Figure 3 Difference in estimated state probabilities, $\pi^1(t)–\pi^2(t)$, after entering long-term full-time sickness absence (SA), where superscript 1 denotes having the Inclusive Working Life Agreement (IA) group and superscript 0 denotes not having IA. Estimates were adjusted for confounding using inverse probability of treatment (IPT)weighting. Faded areas are 95 % confidence intervals based on 1000 bootstrap samples.

For peer review only - http://bmjopen.bmj.com/site/about/guidelines.xhtml

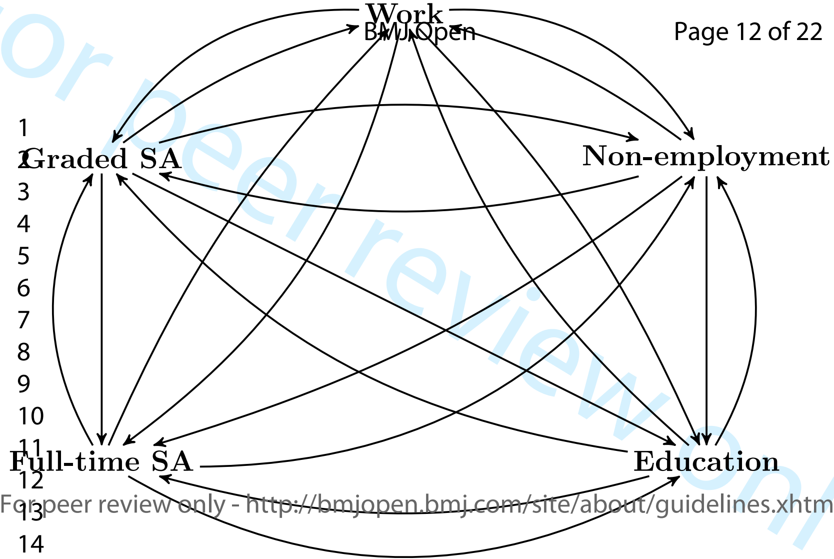

For peer review only - http://bmjopen.bmj.com/site/about/guidelines.xhtml

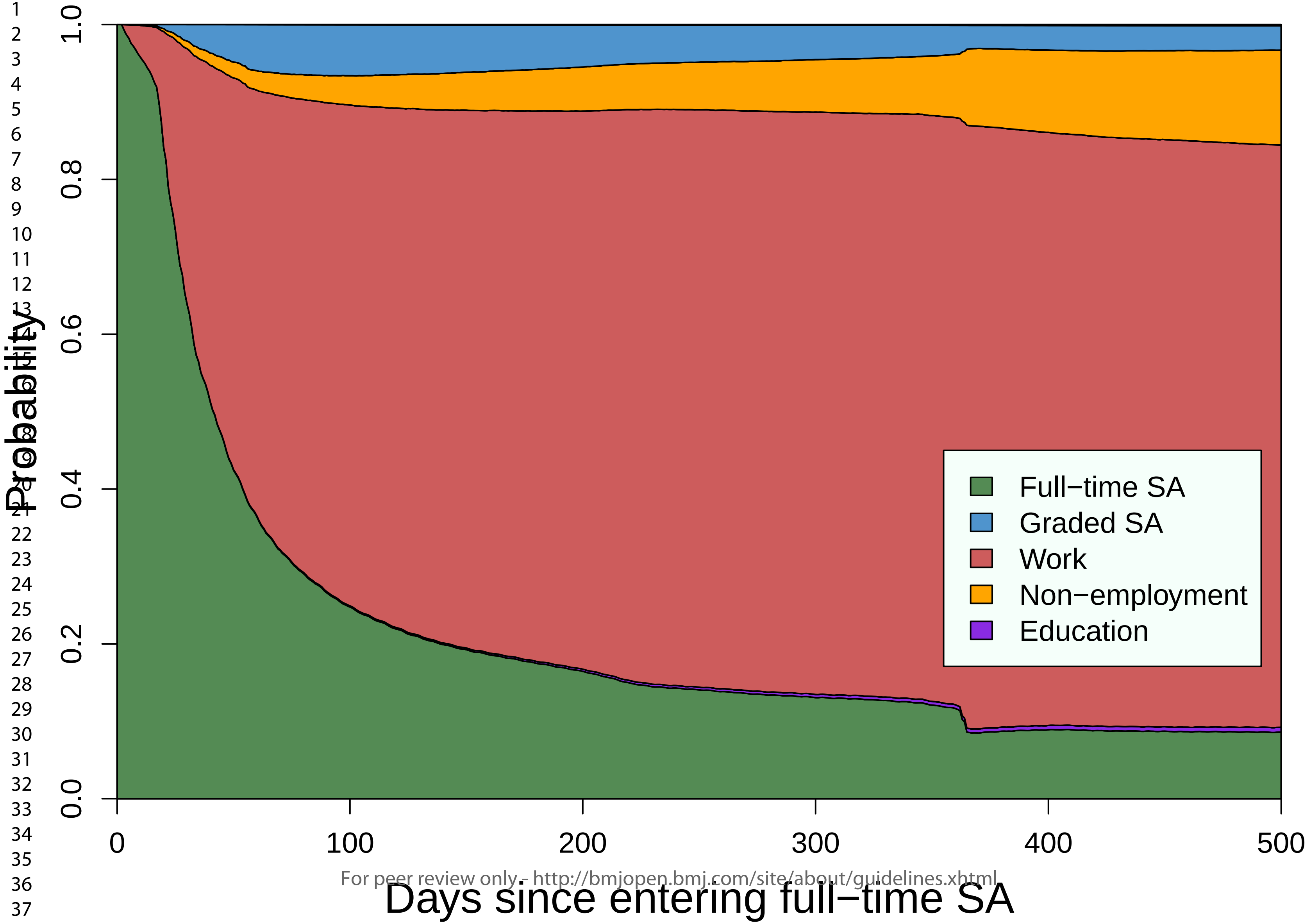

Probability
1.0
0.8
0.6
0.4
0.2
0.0
0
100
200
300
400
500
Days since entering full−time SA
Full−time SA
Graded SA
Work
Non−employment
Education

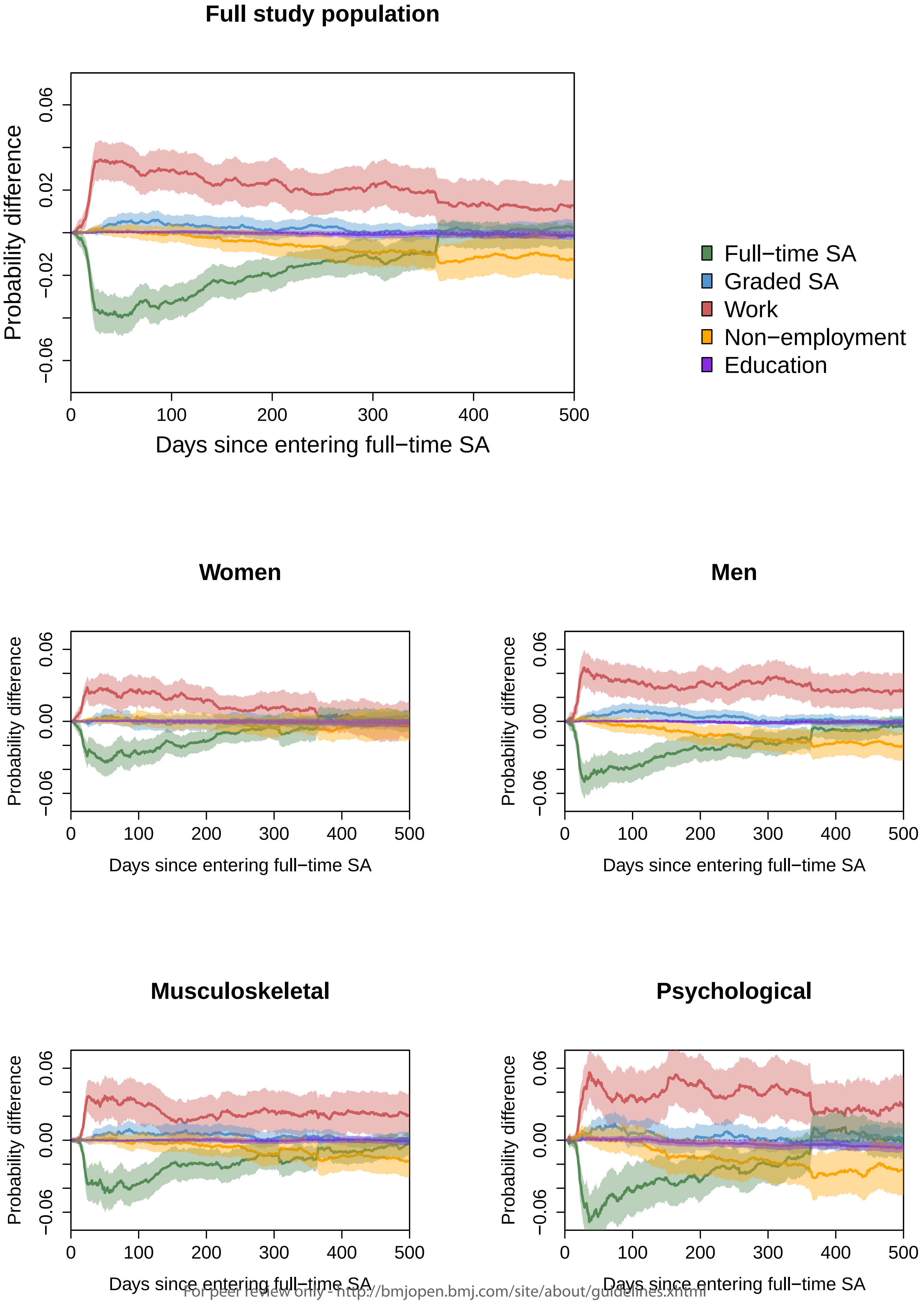
Full study population
Probability difference
0.06
0.02
−0.02
−0.06
0
100
200
300
400
500
Days since entering full−time SA
Full−time SA
Graded SA
Work
Non−employment
Education
Women
Men
Musculoskeletal
Psychological
0.00

An initiative for a more inclusive working life and its effect on return-to-work after sickness absence – a multi-state longitudinal cohort study

Supplementary Material

S.1 Estimands and statistical methods

A multi-state model can be seen as a stochastic process $\{X(t) \mid t \geq 0\}$, taking values from a set of possible states $S = \{1, 2, 3, ..., S\}$ [1, 2, 3]. In the present study, S = 6, and *t* was days since inclusion. The model is governed by transition intensities $\alpha_{jk}(t)$, referring to the probability of a jump to state *k* from state *j* within a small time interval Δt, divided by Δt, as Δt approaches 0:

$$\alpha_{jk}(t) = \lim_{\Delta t \to 0} \frac{\Pr(X(t + \Delta t) = k \mid X(t-) = j)}{\Delta t}$$

for all j,k in S, where j ≠ k.

When $\boldsymbol{A}(t)$ is the matrix of cumulative transition intensities with elements $A_{jk}(t) = \int_0^t \alpha_{jk}(s)\, ds$, for $j \neq k$ and $A_{jj}(t) = -\sum_{j \neq k} A_{jk}(t)$, the state probabilities $\boldsymbol{\pi}(t)$ with elements $\pi_j(t) = \Pr(X(t) = j)$ are found by

$$\widehat{\boldsymbol{\pi}}(t) = \boldsymbol{\pi}(0) \prod_{(0,t]} (\mathbf{I} + \Delta \boldsymbol{A}(u)) \tag{1}$$

where $\boldsymbol{\pi}(0)$ is the initial state distribution at time zero. In settings with time-to-event data, right censoring and no covariates, the elements of $\boldsymbol{A}(t)$ can be estimated using the Nelson-Aalen estimator [4]. State probabilities (also known as state occupational probabilities) are a simple and intuitive quantities for multi-state outcomes and can be seen as an extension of regular survival probabilities (e.g., from Kaplan-Meier curves).

To assess the effect of the Norwegian Inclusive Working Life Agreement (IA), and the within measures for preventing and reducing SA, we sought to identify the average treatment effect (ATE), as the difference in state probabilities

$$\theta(t) = \pi_j^1(t) - \pi_j^0(t) \tag{2}$$

for all j ∈ S, where $\boldsymbol{\pi}^a(t)$ are the counterfactual state probabilities if everyone in the study population was fixed to IA status *a* (taking the values 1 and 0 for IA and non-IA respectively).

From the state probabilities we calculated

$$\theta = \int_0^{365} \pi_j^1(t)\, dt - \int_0^{365} \pi_j^0(t)\, dt \tag{3}$$

representing the difference in expected length of stay (ELOS) in state j, over the first year, for the situations where everyone was fixed to $a = 1$ and $a = 0$.

For peer review only - http://bmjopen.bmj.com/site/about/guidelines.xhtml

The estimands in formula (2) and (3) can be identified under conditional exchangeability (no unmeasured confounding), positivity and consistency [5]. Adjustment for confounding was made by applying baseline inverse probability of treatment (IPT) weights [5], also known as propensity score weights, while fitting hazard models for every possible transition in the model.

More precisely, we fitted a weighted additive hazards regression model [4] on the form

$$\alpha_{jk}(t\,|a) = \beta_{jk0}(t) + \boldsymbol{\beta}_{jk}^{T}(\mathrm{t})a$$

for all possible transitions jk, where $\boldsymbol{\beta}^{\mathrm{T}}$ (t) are time-varying regression functions and $\beta_{jk0}(t)$ is the baseline j → k transition intensity. For each observation we applied the baseline inverse probability of treatment (IPT) weight $\widehat{w}_i$, for the corresponding individual i. Estimates $\widehat{\boldsymbol{B}}_{jk}(t)$of the cumulative regression functions

$$\boldsymbol{B}_{jk}(t) = \left[\int_0^t \beta_{jk0}(u)du\,, \int_0^t \beta_{jk}^{T}(u)du\right]^T$$

are found using least squares techniques [4]. For the two possible values of a, we then found the cumulative transition intensity matrices by

$$\hat{A}_{jk}^{a}(t) = \hat{A}_{jk}(t|a) = \widehat{\boldsymbol{B}}_{jk}^{T}(t)\boldsymbol{a}^{*}$$

where $\boldsymbol{a}^{*} = (1, a)^{T}$, which again was plugged into the formula in (1), and thus identify the estimands in formula (2) and (3).

To calculate the IPT weights $\widehat{w}_i$, we modelled IA status A, given baseline confounders $\mathbf{Z}$ in a logistic regression model

$$logit\,\Pr(A|\,\mathbf{Z}) = \beta^{T}\mathbf{Z}$$

Using this model, for each individual i, the conditional probability of having their assigned IA status a_i given their covariate values z_i at baseline, $\Pr(a_i \mid \boldsymbol{z}_i)$ were estimated and used to calculate the (stabilized) IPT weight [5]

$$\widehat{w}_i = \frac{\Pr(a_i)}{\Pr(a_i\,|\boldsymbol{z}_i)}$$

where $\Pr(a_i)$ is the sample proportion with IA status equal to a_i at baseline.

S.2 Sensitivity analysis 1: Using subsets of adjustment variables

As a first sensitivity analysis, we compared the results from the main analysis with results from a series of analyses adjusting for all possible subsets of the main adjustment set, including the empty set – in total 256 models. Results are shown in Supplementary Figure 1.

In the left panel of Supplementary Figure 1, the unadjusted differences in state probabilities are seen to be almost twice as big as after confounder adjustment using IPT weighting. While in the right panel, we see how including smaller and less complete sets of confounders can lead to shifts in both directions compared to the main model.

For peer review only - http://bmjopen.bmj.com/site/about/guidelines.xhtml

S.3 Sensitivity analysis 2: Adjusting for additional covariates measured for men during military

Most of the men included in the study underwent military conscript examinations at the age of 18. Here they measured, among other things, IQ score, BMI, physical stamina score and a rough assessment of military eligibility (physical and mental). These data were available to us from the Norwegian Armed Forces Personnel Data Base. As these variables were not measured for all the individuals in the cohort, especially women, they were not adjusted for in the primary analyses. However, as a sensitivity analysis, we compared weighed analysis for men not taking and taking these variables into account. The results are shown in the left panel in Supplementary Figure 2. The plot shows no visible differences. In fact, the absolute differences in effect estimates came after the third – fourth decimal. We also tried adding conscript variables in an alternative analysis where the variables for education and workplace industry from the main analysis were left out. Here we were able to detect visible differences in effect estimates from the plot. Results of this analysis are shown in the right panel of Supplementary Figure 2.

S.4 Sensitivity analysis 3: Pre IA (1997-1999) multi-state histories as a negative control

Individual multi-state histories are also available for individuals in the cohort before IA was introduced and can be utilised as a negative outcome control [6]. The question of interest is then whether these earlier multi-state histories differed between those with later IA and those without, and if the IPT weights used sufficiently adjusted for such a difference. We therefore considered state histories during the period from 1st of January 1997 to 31st of December 1999, where we compared both the crude difference in state probabilities for the same intervention groups as in the main analysis, and a weighted analysis using the IPT weights of the main analysis. The results are found in Supplementary Figure 3. Results showed that the later IA groups differed between 1997 and 2000 in terms of education probability (IA group about 4 percentage points more likely to be in education) and work probability (non-IA group about 3 percentage points more likely to be in work). For non-employment there were only small differences. The probabilities for full-time or graded SA did not differ much, as one would expect due to the restriction of no history of SA one year before inclusion. However, after applying the IPT weights from the main analysis, also the differences in work histories that were there disappeared. This indicates that the confounding adjustment through these weights sufficiently covered differences in earlier work life (all states) histories.

S.5 Sensitivity analysis 4: Analysis of a limited selection of industries

From the information in Table 2 in the main article, it appeared that a large proportion of IA companies were in industries where public workplaces are typically overrepresented, such as education and health services. Information on public vs private workplace was not available for the study. In this section, we performed a separate analysis for a limited selection of industries, assumed to have had a majority of private workplaces, namely wholesale and retail, construction, manufacturing, commercial services and transport and storage. Results from these analyses are found in Supplementary Figure 4. We found a higher effect of IA within this subgroup, more akin to the effect seen in men in the main article.

For peer review only - http://bmjopen.bmj.com/site/about/guidelines.xhtml
For peer review only

References

[1] Hougaard P. Multi-state models: a review. Lifetime Data Analysis. 1999;5(3):239–264.

[2] Andersen PK, Keiding N. Multi-state models for event history analysis. Stat Methods Med Res. 2002;11(2):91–115.

[3] Putter H, Fiocco M, Geskus RB. Tutorial in biostatistics: competing risks and multi-state models. Stat Med. 2007;26(11):2389–2430.

[4] Aalen OO, Borgan Ø, Gjessing H. Survival and event history analysis: a process point of view. New York, NY: Springer; 2008.

[5] Hernan M, Robins JM. Causal Inference. Boca Raton: Chapman & Hall/CRC; 2020.

[6] Lipsitch M, Tchetgen ET, Cohen T. Negative controls: a tool for detecting confounding and bias in observational studies. Epidemiology (Cambridge, Mass). 2010;21(3):383.

For peer review only

Tables

Supplementary Table 1 Total number of transitions made by the study population of n = 187 930 individuals, over 90 231 405 person days, during individual follow-up of up to 500 days after entering full-time sickness absence (SA).

**FROM \ TO**	Full-time SA	Work	Graded SA	Non-employment	Education	Death
Full-time SA	0	197 774	32939	18760	492	163
Work	64 888	0	18562	37074	2003	78
Graded SA	9127	35254	0	1853	49	0
Non-employment	5902	28468	750	0	1015	34
Education	131	1526	31	737	0	0
Death	0	0	0	0	0	0

For peer review only - http://bmjopen.bmj.com/site/about/guidelines.xhtml

Figures

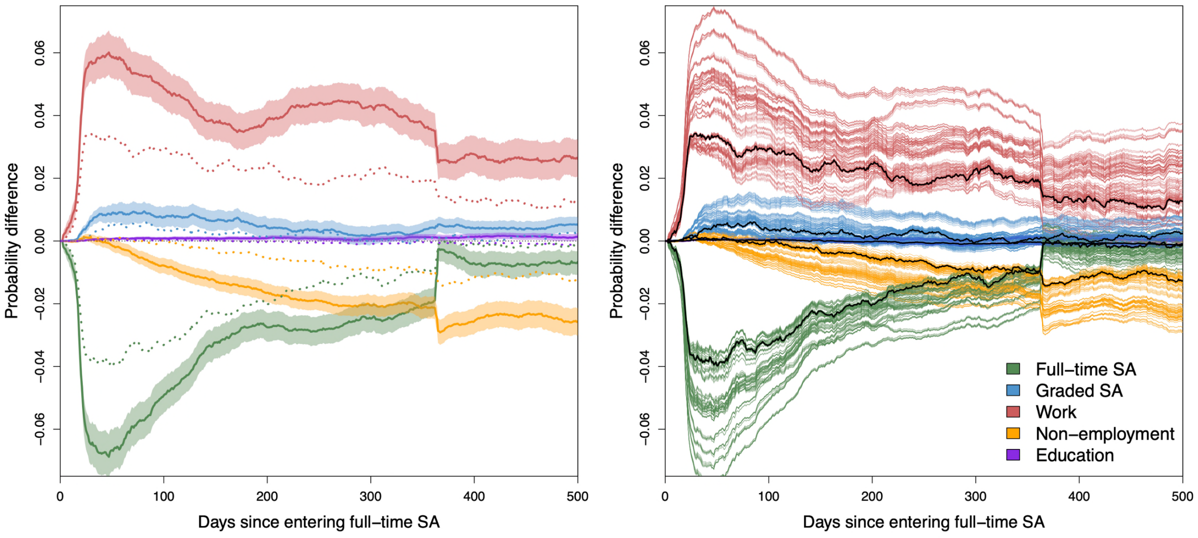

Supplementary Figure 1: Differences in estimated state probabilities, $\hat{\boldsymbol{\pi}}^1(t) - \hat{\boldsymbol{\pi}}^0(t)$, for the full study population, after entering long-term full-time sickness absence (SA), where superscript 1 denotes having the Inclusive Working Life Agreement (IA) and superscript 0 denotes not having IA. Faded areas are 95 % confidence intervals based on 1000 bootstrap samples. **Left:** The full-drawn lines are unadjusted estimates, while the dotted lines show the inverse probability of treatment (IPT) weighted estimates from the main article. **Right:** The black lines illustrate the IPT weighted estimates from the primary analysis in the main paper and colored lines are IPT weighted estimates from using different subsets of the main adjustment set when constructing the weights.

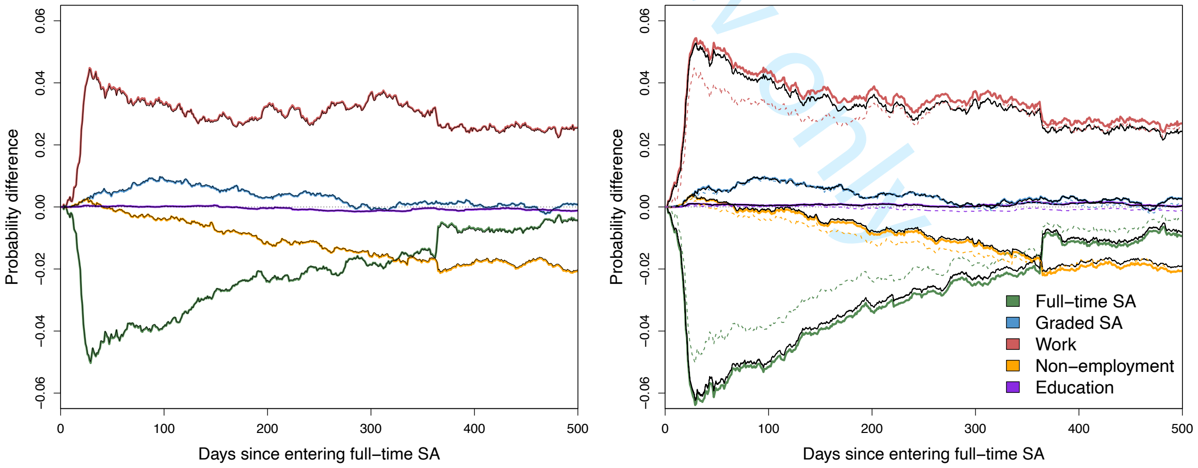

Supplementary Figure 2: Difference in estimated state probabilities, $\hat{\boldsymbol{\pi}}^1(t) - \hat{\boldsymbol{\pi}}^0(t)$, for men who completed military conscript examinations, after entering long-term full-time sickness absence (SA), where superscript 1 denotes having the Inclusive Working Life Agreement (IA) and superscript 0 denotes not having IA. **Left:** The colored lines are estimates adjusted for confounding by inverse probability of treatment (IPT) weighting using the main confounder set. Black lines show the estimates from when conscript variables IQ score, BMI, physical stamina test and military eligibility test were also adjusted for. **Right:** The black lines show estimates where also conscript variables IQ score, BMI, physical stamina test and military eligibility test were adjusted for but leaving out the covariates for education and workplace industry. Full drawn

For peer review only - http://bmjopen.bmj.com/site/about/guidelines.xhtml

colored lines are estimates adjusted for confounding by IPT weighting, leaving out education and workplace industry from the confounder set. Dotted colored lines are original estimates adjusted for confounding using the full main adjustment set.

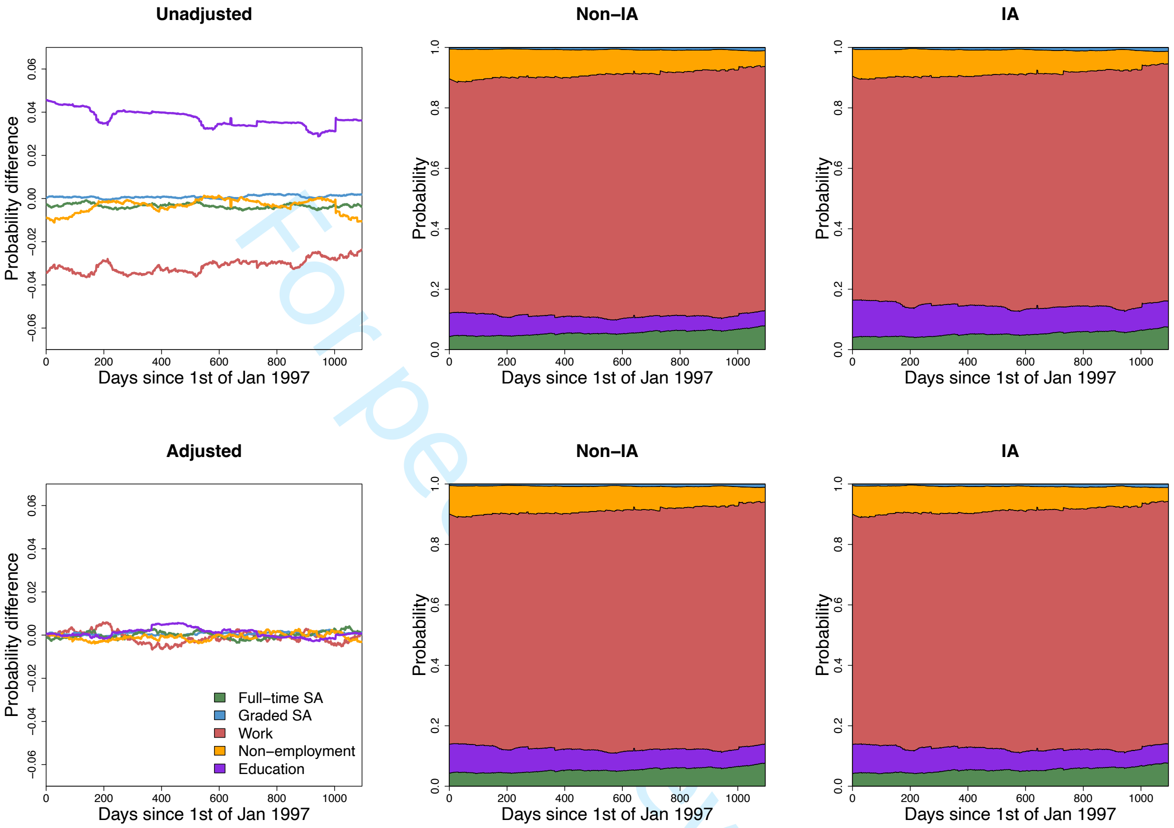

Supplementary Figure 3: The left column shows differences in state probabilities, $\hat{\boldsymbol{\pi}}^1(t) - \hat{\boldsymbol{\pi}}^0(t)$, where superscript 1 denotes having the Inclusive Working Life Agreement (IA) and superscript 0 denotes no IA. The middle and right column show state occupation probabilities for no IA and IA respectively. The top row shows unadjusted estimates, while the bottom row shows estimates adjusted for confounding using inverse probability of treatment (IPT) weighting.

For peer review only - http://bmjopen.bmj.com/site/about/guidelines.xhtml

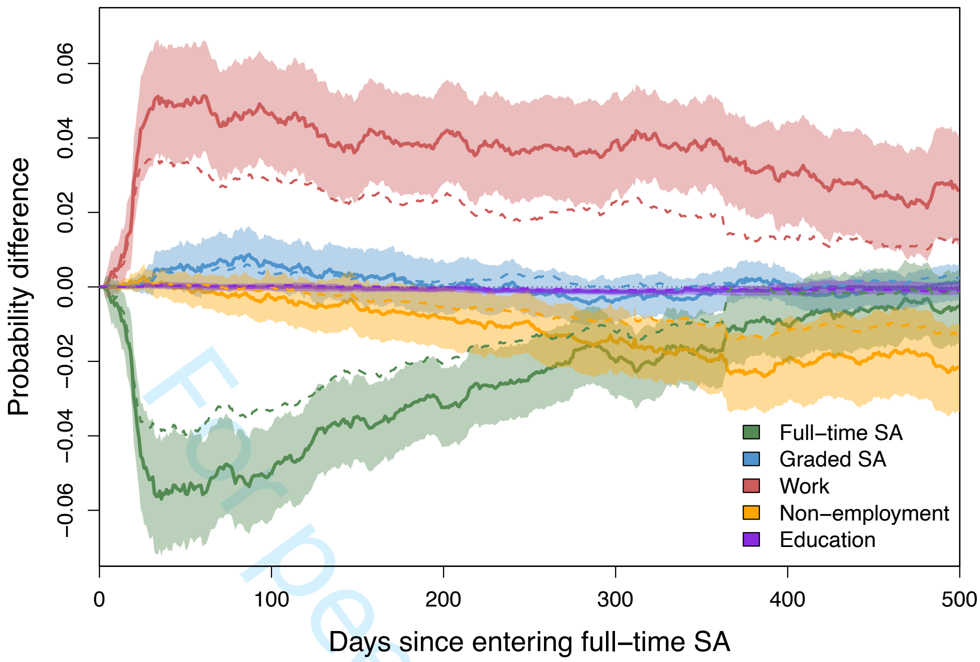

Supplementary Figure 4: Difference in estimated state probabilities, $\hat{\pi}^1(t) - \hat{\pi}^0(t)$, after entering long-term full-time sickness absence (SA), where superscript 1 denotes having the Inclusive Working Life Agreement (IA) and superscript 0 denotes not having IA. Estimates are adjusted for confounding using inverse probability of treatment (IPT) weighting. Faded areas are 95 % confidence intervals based on 1000 bootstrap samples. Full-drawn lines are estimates for the limited selection of industries, wholesale and retail, construction, manufacturing, commercial services and transport and storage, while the stippled lines show the estimates from the primary analysis (all industries).

For peer review only - http://bmjopen.bmj.com/site/about/guidelines.xhtml

STROBE Statement—Checklist of items that should be included in reports of ***cohort studies***

	Item No	Recommendation	Page No
**Title and abstract**	1	(*a*) Indicate the study's design with a commonly used term in the title or the abstract<br>(*b*) Provide in the abstract an informative and balanced summary of what was done and what was found	a) 1<br>b) 1
**Introduction**			
Background/rationale	2	Explain the scientific background and rationale for the investigation being reported	1-2
Objectives	3	State specific objectives, including any prespecified hypotheses	1-2
**Methods**			
Study design	4	Present key elements of study design early in the paper	2
Setting	5	Describe the setting, locations, and relevant dates, including periods of recruitment, exposure, follow-up, and data collection	2-3
Participants	6	(*a*) Give the eligibility criteria, and the sources and methods of selection of participants. Describe methods of follow-up<br>(*b*) For matched studies, give matching criteria and number of exposed and unexposed	a) 2
Variables	7	Clearly define all outcomes, exposures, predictors, potential confounders, and effect modifiers. Give diagnostic criteria, if applicable	2-3
Data sources/ measurement	8*	For each variable of interest, give sources of data and details of methods of assessment (measurement). Describe comparability of assessment methods if there is more than one group	2-3
Bias	9	Describe any efforts to address potential sources of bias	2-3
Study size	10	Explain how the study size was arrived at	2
Quantitative variables	11	Explain how quantitative variables were handled in the analyses. If applicable, describe which groupings were chosen and why	2-3
Statistical methods	12	(*a*) Describe all statistical methods, including those used to control for confounding<br>(*b*) Describe any methods used to examine subgroups and interactions<br>(*c*) Explain how missing data were addressed<br>(*d*) If applicable, explain how loss to follow-up was addressed<br>(*e*) Describe any sensitivity analyses	a) 3-4, S1-2<br>b) 3<br>c) 2<br>e) 3
**Results**			
Participants	13*	(a) Report numbers of individuals at each stage of study—eg numbers potentially eligible, examined for eligibility, confirmed eligible, included in the study, completing follow-up, and analysed<br>(b) Give reasons for non-participation at each stage<br>(c) Consider use of a flow diagram	a) 2<br>b) 2
Descriptive data	14*	(a) Give characteristics of study participants (eg demographic, clinical, social) and information on exposures and potential confounders<br>(b) Indicate number of participants with missing data for each variable of interest<br>(c) Summarise follow-up time (eg, average and total amount)	a) 4<br>c) 4,S4
Outcome data	15*	Report numbers of outcome events or summary measures over time	sup4

For peer review only

For peer review only - http://bmjopen.bmj.com/site/about/guidelines.xhtml

Main results	16	(*a*) Give unadjusted estimates and, if applicable, confounder-adjusted estimates and their precision (eg, 95% confidence interval). Make clear which confounders were adjusted for and why they were included<br>(*b*) Report category boundaries when continuous variables were categorized<br>(*c*) If relevant, consider translating estimates of relative risk into absolute risk for a meaningful time period	a) 4,<br>b) 4
Other analyses	17	Report other analyses done—eg analyses of subgroups and interactions, and sensitivity analyses	6-7 S2-7
**Discussion**			
Key results	18	Summarise key results with reference to study objectives	7-8
Limitations	19	Discuss limitations of the study, taking into account sources of potential bias or imprecision. Discuss both direction and magnitude of any potential bias	8
Interpretation	20	Give a cautious overall interpretation of results considering objectives, limitations, multiplicity of analyses, results from similar studies, and other relevant evidence	8
Generalisability	21	Discuss the generalisability (external validity) of the study results	8
**Other information**			
Funding	22	Give the source of funding and the role of the funders for the present study and, if applicable, for the original study on which the present article is based	8

*Give information separately for exposed and unexposed groups.

Note: An Explanation and Elaboration article discusses each checklist item and gives methodological background and published examples of transparent reporting. The STROBE checklist is best used in conjunction with this article (freely available on the Web sites of PLoS Medicine at http://www.plosmedicine.org/, Annals of Internal Medicine at http://www.annals.org/, and Epidemiology at http://www.epidem.com/). Information on the STROBE Initiative is available at http://www.strobe-statement.org.

For peer review only

BMJ Open

An initiative for a more inclusive working life and its effect on return-to-work after sickness absence – a multi-state longitudinal cohort study

Journal:	*BMJ Open*
Manuscript ID	bmjopen-2022-062558.R1
Article Type:	Original research
Date Submitted by the Author:	06-Sep-2022
Complete List of Authors:	Hoff, Rune; Oslo University Hospital<br>Maltzahn, Niklas; Oslo University Hospital; University of Oslo, Department of Biostatistics<br>Hasting, Rachel; STAMI<br>Merkus, Suzanne L; STAMI<br>Undem, Karina; STAMI<br>Kristensen, Petter; STAMI, Occupational Medicine and Epidemiology<br>Mehlum, Ingrid; STAMI, Occupational Medicine and Epidemiology; University of Oslo, Department of Community Medicine and Global Health<br>Gran, Jon; University of Oslo Faculty of Medicine, Department of biostatistics; Oslo University Hospital
**Primary Subject Heading**:	Occupational and environmental medicine
Secondary Subject Heading:	Occupational and environmental medicine
Keywords:	OCCUPATIONAL & INDUSTRIAL MEDICINE, STATISTICS & RESEARCH METHODS, Health policy < HEALTH SERVICES ADMINISTRATION & MANAGEMENT

SCHOLARONE™ Manuscripts

I, the Submitting Author has the right to grant and does grant on behalf of all authors of the Work (as defined in the below author licence), an exclusive licence and/or a non-exclusive licence for contributions from authors who are: i) UK Crown employees; ii) where BMJ has agreed a CC-BY licence shall apply, and/or iii) in accordance with the terms applicable for US Federal Government officers or employees acting as part of their official duties; on a worldwide, perpetual, irrevocable, royalty-free basis to BMJ Publishing Group Ltd ("BMJ") its licensees and where the relevant Journal is co-owned by BMJ to the co-owners of the Journal, to publish the Work in this journal and any other BMJ products and to exploit all rights, as set out in our licence.

The Submitting Author accepts and understands that any supply made under these terms is made by BMJ to the Submitting Author unless you are acting as an employee on behalf of your employer or a postgraduate student of an affiliated institution which is paying any applicable article publishing charge ("APC") for Open Access articles. Where the Submitting Author wishes to make the Work available on an Open Access basis (and intends to pay the relevant APC), the terms of reuse of such Open Access shall be governed by a Creative Commons licence – details of these licences and which Creative Commons licence will apply to this Work are set out in our licence referred to above.

Other than as permitted in any relevant BMJ Author's Self Archiving Policies, I confirm this Work has not been accepted for publication elsewhere, is not being considered for publication elsewhere and does not duplicate material already published. I confirm all authors consent to publication of this Work and authorise the granting of this licence.

For peer review only

An initiative for a more inclusive working life and its effect on return-to-work after sickness absence – a multi-state longitudinal cohort study

Rune Hoff, PhD[1*], Niklas Maltzahn, MSc[1,2], Rachel Hasting, MSc[3], Suzanne L Merkus, PhD[4], Karina Undem, MSc[3], Petter Kristensen, PhD[3], Ingrid Sivesind Mehlum, PhD[3,5] and Jon Michael Gran, PhD[2,1]

[1*]Oslo Centre for Biostatistics and Epidemiology, Oslo University Hospital, Norway.
[2]Oslo Centre for Biostatistics and Epidemiology, Department of Biostatistics, Institute of Basic Medical Sciences, University of Oslo, Norway.
[3]Research Group for Occupational Medicine and Epidemiology, National Institute of Occupational Health, Oslo, Norway. [4]Research Group for Work Psychology and Physiology, National Institute of Occupational Health, Oslo, Norway [5]Department of Community Medicine and Global Health, Institute of Health and Society, University of Oslo, Norway.

*Corresponding author Rune Hoff. E-mail: rune.hoff@medisin.uio.no, Postal address: Oslo University Hospital, Sogn Arena, Oslo Centre for Biostatistics and Epidemiology, Pb 4950 Nydalen, 0424 Oslo, Norway.

For peer review only

Abstract

Objectives To reduce sickness absence (SA) and increase work participation, the tripartite Agreement for a More Inclusive Working Life (IA) was established in Norway in 2001. IA companies have had access to several measures to prevent and reduce SA. Our aim in this paper was to estimate the average effect of having access to IA at the time of entering a first SA on later return-to-work (RTW) and on time spent in other work-related states. A secondary objective was to study how effects varied between women and men, and individuals with SA due to either musculoskeletal or psychological diagnoses.

Design Population based observational multi-state longitudinal cohort study.

Participants Each individual born in Norway 1967-1976 who entered full-time SA during 2004-2011, with limited earlier SA, was included (n = 187 930).

Outcome measures Individual multi-state histories containing dated periods of work, graded SA, full-time SA, non-employment and education.

Methods Individual characteristics and detailed longitudinal records of SA, work and education were obtained from population-wide registries and analysed in a multi-state model with 500 days of follow-up. The effect of IA was assessed by estimating differences in state probabilities over time, adjusted for confounders using inverse probability weighting.

Results IA increased the probability of work after SA, with largest difference between groups after 29 days (3.4 percentage points higher (95% confidence interval: 2.5 – 4.3)). Differences in one-year expected length of stay was 8.4 additional days (4.9 – 11.9) in work, 7.6 (4.8 – 10.3) fewer days in full-time SA and 1.6 (-0.2 – 3.4) fewer days in non-employment. Similar trends were found within subgroups by sex, musculoskeletal and psychological diagnoses. The robustness of the findings was studied in sensitivity analyses.

Conclusion Measures to prevent and reduce SA, as given through IA, were found to improve individuals' RTW after entering SA.

Keywords: Workplace initiative, occupational health, working life policy

Word count: 4852

Strengths and limitations of this study

- This is the first study on the effects of the Norwegian population-wide initiative to increase work-participation that analyses a large dataset of longitudinal multi-state outcomes.
- The data exhibit a high level of completeness and include also detailed covariate information.
- Limitations of the study are that we only had access to observational data and that IA was not randomised.

Introduction

Sickness absence (SA) from work is an important long-term health outcome for the individual, as well as a social and financial burden for society. [1– 3] It has been shown that while in SA, individuals are at greater risk of a more permanent exit from working life. [4, 5] SA increased greatly in Norway between 1995 and 2000, mainly due to an increase in long-term SA (more than 16 calendar days). The yearly average days of SA per worker in private workplaces, increased from 8.8 days to 12.9 days in the same period.[6] It should be noted that this increase came after a period of reduction in SA,[7] and that around one third of the increase can be explained by changes in the size and age of the workforce.[6]

In 2001, the Norwegian Government reached out to major partners in working life, representing both workers and employers, with the intention of reducing SA and prolonging working life. This resulted in the tripartite Agreement for a More Inclusive Working Life (IA), which is still active, and included three operative goals: i) reduce SA by at least 20 %, ii) increase employment for individuals with functional limitations and iii) increase the average retirement age. [6] By 2004, 55% of workers were in IA companies, i.e., companies that signed a cooperation agreement with the Norwegian Labour and Welfare Administration (NAV) and the employee representatives in the company, to cooperate systematically to achieve more inclusive workplaces. [8]

IA companies and their employees have access to several measures to reduce and prevent SA. These include special regulations and support from working life centres that were established regionally by NAV. The support from these centres can be comprehensive, including tailored support for employees with health problems. [9] The measures included the possibility for employees in SA to use so-called active sickness benefits, where they may return to work and perform modified tasks while still receiving payments from NAV. Employees in IA companies are also allowed to have longer self-certified SA (up to eight consecutive calendar days, instead of three, and a total of 24 annual days, instead of 12). The IA companies may receive refunds for measures related to aiding employees in returning to work. If it is impossible for an employee to return to the same position they had before SA, the IA companies have agreed to cooperate with NAV in assisting in retraining so that the employee can continue to work in the same company.

Since implemented, IA has not met the goals regarding SA, and systematic evaluations, although sparse, suggest limited or no effect on the risk of experiencing SA. [8, 10, 11, 12] Studies of other general workplace interventions have found that they were generally not effective in reducing SA. [13] However, there are studies suggesting effects of workplace interventions on RTW. [12, 14]

The main objective of this study was to estimate the average effect of having access to IA at the time of entering a first SA on later RTW and on time spent in other work-related states for the following 500 days. To analyse such individual outcome trajectories in more detail than have been done before, we utilised a multi-state model, [15] capturing individuals' movement between the states of full-time SA, graded SA, work, non-employment and education over time. Inverse probability of treatment (IPT) weighting was used to adjust for confounding between IA and multi-state outcomes. [16] The effect of IA has not previously been studied in detail at a population level using such methods. A secondary objective was to study how effects varied between women and men, and individuals with SA due to either musculoskeletal or psychological diagnoses.

Methods

Design, participants and exposure

For peer review only - http://bmjopen.bmj.com/site/about/guidelines.xhtml

To meet the study objectives, we utilised observational longitudinal registry data from a cohort consisting of all individuals born in Norway between 1967 and 1976 (626 928 individuals), as registered by the Medical Birth Registry of Norway. The same cohort is described in greater detail by Kristensen and Bjerkedal. [17] Given limited earlier SA (no SA in 2003), individuals were included in the analysis if they entered full-time SA (>16 calendar days, counted from the first day), between January 1, 2004, when IA was properly implemented, and December 31, 2010. In total this comprised 187 930 persons. Baselines were set to the dates of the initial SA episode for all individuals, who simultaneously had their IA exposure (yes/no) recorded, based on the IA status of the company they worked for, and were followed for 500 days or until death, right-censoring or administrative censoring on January 1, 2011. The choice of 500 days was a pragmatic choice, meant to cover the first full year and a short period beyond. The immediate period after the first year is of interest, as welfare policies impose a change in benefits received after a full year of receiving sick leave benefits. Moreover, for even longer follow-up periods, individuals' IA status would be more likely to change, making results from an analysis based on baseline IA status more difficult to interpret.

Patient and public involvement

Patients or the public were not involved in the design, or conduct, or reporting, or dissemination plans of our research.

Outcomes and confounders

The individual multi-state histories are described by dated transitions between periods of work, graded SA, full-time SA, non-employment, education and death, referred to as states, as shown in Figure 1. Multi-state models can be seen as extensions of standard time-to-event models with only two states, such as alive and dead. In multi-state models, individuals are allowed to have several transitions while remaining under observation. In our application, individuals started in full-time SA and could move back and forth between transient states at any time during the 500 days follow-up period, and for example have multiple spells of full-time SA. The exception is death, which is a so-called absorbing state, where further transitions are not possible.

As the IA Agreement is not randomly assigned to individuals, the association between IA and the individual multi-state histories may be confounded by various characteristics that are different between individuals and workplaces with and without IA and need to be adjusted for. For this purpose, information was collected at individual level on the assumed confounders recorded at individuals' baseline: age, sex, education level, calendar year, company size, geographical region and type of industry. Education level was the individual's highest attained degree, separated into four categories: lower secondary, upper secondary, undergraduate and graduate. Type of industry covered 20 categories in the Statistical Classification of Economic Activities in the European Community. [18] Company size was based on yearly records of number of employees, and company region comprised southern, eastern, western, middle, and northern parts of Norway.

Data sources and data preparation

All the data were collected from Statistics Norway's event database of employment and welfare (FD-Trygd), The National Education Database and the registers of the Norwegian Labour and Welfare Administration. The data contained individual and company characteristics acting as potential baseline confounders, individual status of the main intervention (IA status), dated records of all individuals' work (with taxable income), SA (>16 calendar days) and education. From the records of work, SA and education, we generated individual multi-state outcome histories, or trajectories, over a period of 500 days, with the possible states of full-time SA, graded SA, work, education and non-employment.

Individuals were included in the study on the date of their first full-time SA episode after the first of January 2004, given that they had not been in SA the previous year. All the baseline confounders were set to the values as recorded on each individual inclusion date. IA status was set to the IA status of the individual's company at the time of inclusion. Individuals working for more than one company during follow-up were seen as having IA if either of the workplaces had IA. For this study, SA included periods eligible for either of three different types of benefits in the Norwegian welfare system: sick leave benefit, work assessment allowance (medical or vocational rehabilitation benefits) and disability pension, all attainable as full-time (100%) or graded (< 100%). Work referred to paid employment and included holidays and parental leave, while education covered admittance to officially licensed education programs. Periods without SA, work or education were considered as non-employment if they had later records of SA, work or education. Loss to follow-up without any later records

For peer review only - http://bmjopen.bmj.com/site/about/guidelines.xhtml

were treated as right-censoring. Whenever different types of records occurred simultaneously on the same dates for an individual, different records had precedence in the following order, from most to least important: SA, work and lastly education.

Subgroup and sensitivity analyses

In addition to the main analysis, separate analyses were done for women and men, and for diagnosis group associated with initial SA (musculoskeletal or psychological). Musculoskeletal diagnoses consisted of ICPC-2 (International Classification of Primary Care) [19] codes starting with L, while psychological diagnoses referred to codes starting with P.

We conducted five sensitivity analyses. First, we compared results adjusting for subsets of the confounders, to see how omitted variables in the adjustment set could bias results. In the second, we adjusted for additional potential confounders available for a male subgroup of the study population, describing IQ, physical stamina, BMI and eligibility for military service. In the third, we used pre-baseline longitudinal outcomes between 1997-1999, as negative outcome controls. [20] If confounding was sufficiently adjusted for, state transition histories before baseline should be similar across IA groups, after weighting with the same IPT weights as in the main analysis. In the fourth, we analysed a subset of industries with mostly private companies. Lastly, since our study population were in the age span 28-43 at entry into the study, and a substantial part (20,355) of the initial SA cases was in connection with pregnancy, childbearing and family planning (ICPC-2 codes starting with W), an analysis where these cases were removed was conducted.

Although type of industry is adjusted for in the main analysis, we also created sequence plots of individuals' multi-state histories within five different industry sectors for descriptive purposes. These plots illustrate typical patterns of multi-state trajectories at the individual level, over the 500 days of follow-up. We also looked at gender balance within the largest industries of our study population for discussion purposes.

A closer description of these analyses is found in the supplementary material.

Estimands and statistical methods

The individual multi-state outcomes were analysed using hazard based multi-state models for time-to-event data, [15] by first fitting hazard models for each transition and then estimating state probabilities for all states in Figure 1. The model allows individuals to move, possibly back and forth, between states over time according to the possible transitions illustrated in the figure. By state probabilities we here mean the probability of being in a particular state at a given time-point, which can be seen as an extension of regular survival probabilities (e.g., Kaplan-Meier curves). To assess the effect of IA, we sought to identify the average treatment effect, as the difference in state probabilities $\theta(t) = \pi^1(t) - \pi^0(t)$, where $\pi^a(t)$ is the adjusted marginal state probability, corresponding to the state probability we would have seen if everyone in the target population was fixed to having IA status equal to a (taking the values 1 and 0 for IA and no IA, respectively). From the state probabilities we also calculated the expected length of stay (ELOS) [21] in the different states over the first year, for the situations where everyone was fixed to $a = 1$ or $a = 0$.

Confounding adjustment was done by calculating baseline stabilized IPT weights, [22] also known as propensity score weights, and then fitting weighted additive hazards regression models [23] for every possible transition in Figure 1, adjusting only for IA status. This corresponds to using weighted Nelson-Aalen estimators for the cumulative hazards separately for the two IA groups. The corresponding state probabilities were then estimated by plugging the transition-specific cumulative hazard estimates into the Aalen-Johansen estimator. The ELOS can be calculated directly from the Aalen-Johansen estimates, as the area under the curve of the corresponding state probabilities over time. To calculate the IPT weights we modelled IA status as a function of the baseline confounders in a logistic regression model. Since IA is an intervention at the company level, we calculated 95% confidence intervals using a clustered bootstrap where the resampling is done on company level.[24]

For a more formal specification of the target estimands and details on the statistical methods, see section S.1 in the supplementary material, and, more generally, Andersen and Keiding [15], Gran et al [16] and Aalen et al [23]. All analyses were performed in R, on the TSD (Services for Sensitive Data) facilities, owned by the University of Oslo. R code is available on GitHub (see Declarations).

For peer review only - http://bmjopen.bmj.com/site/about/guidelines.xhtml

Results

Descriptive statistics

Over a total follow-up of 90 231 405 person days, the 187 930 individuals included made a total of 457 610 transitions, which are summarized in detail in Supplementary Table 1. A summary of the individual characteristics can be found in Table 1. In the second and third column of the latter table, we can see how the measured confounders are distributed differently among the IA and non-IA individuals, with, for example, more women and individuals in larger workplaces having IA. In the fourth and fifth column, however, we see how the measured confounders are distributed in the weighted data, after applying the IPT weights, with the measured confounding characteristics now being well balanced between groups.

Table 1 Descriptive statistics for individuals working in companies with the Inclusive working life Agreement (IA), or without, at the time of inclusion. The table also describes the inverse probability of treatment (IPT) weighted population.

	No IA, n (%)	IA, n (%)	No IA, n (%)	IA, n (%)
	n = 119,363	n = 68,567	*IPT Weighted*	*IPT Weighted*
**Sex**				
Women	57,002 (0.48)	45,139 (0.66)	63,383 (0.53)	37,907 (0.55)
Men	62,361 (0.52)	23,428 (0.34)	55,037 (0.46)	30,885 (0.45)
**Age at inclusion**				
28-32	33,876 (0.28)	16,336 (0.24)	31,722 (0.27)	18,619 (0.27)
33-37	60,561 (0.51)	34,485 (0.50)	60,150 (0.50)	35,121 (0.51)
38-43	24,926 (0.21)	17,746 (0.26)	26,547 (0.22)	15,052 (0.22)
**Inclusion year**				
2004	31,255 (0.26)	12,762 (0.19)	28,556 (0.24)	17,376 (0.25)
2005	25,935 (0.22)	12,671 (0.18)	24,741 (0.21)	14,278 (0.21)
2006	19,812 (0.17)	11,373 (0.17)	19,757 (0.17)	11,169 (0.16)
2007	14,951 (0.13)	9,895 (0.14)	15,307 (0.13)	8,769 (0.13)
2008	11,955 (0.10)	9,094 (0.13)	12,704 (0.11)	7,258 (0.11)
2009	10,082 (0.08)	8,029 (0.12)	11,234 (0.09)	6,366 (0.09)
2010	5,373 (0.05)	4,743 (0.07)	6,121 (0.05)	3,578 (0.05)
**Education**				
Lower secondary	39,162 (0.33)	17,005 (0.25)	35,319 (0.30)	19,753 (0.29)
Upper secondary	52,977 (0.44)	241,37 (0.35)	48,767 (0.41)	28,885 (0.42)
College	23,393 (0.20)	23,754 (0.35)	28,851 (0.24)	17,392 (0.25)
University	3,831 (0.03)	3,671 (0.05)	5,483 (0.05)	2,763 (0.04)
**Industry**				
Agriculture/forestry	1,406 (0.01)	106 (0.00)	963 (0.01)	720 (0.01)
Commercial services	7,015 (0.06)	1,811 (0.03)	5,633 (0.05)	3,381 (0.05)
Construction	12,357 (0.10)	2,763 (0.04)	9,571 (0.08)	4,785 (0.07)
Education	3,959 (0.03)	9,624 (0.14)	9,594 (0.08)	4,941 (0.07)
Scientific service work	5,475 (0.05)	853 (0.01)	4,006 (0.03)	2,120 (0.03)
Electricity supply	387 (0.00)	317 (0.00)	473 (0.00)	289 (0.00)
Entertainment	1,502 (0.01)	379 (0.01)	1,203 (0.01)	944 (0.01)
Financial	1,701 (0.01)	1,189 (0.02)	1,923 (0.02)	1,282 (0.02)
Health and social work	16,658 (0.14)	27,779 (0.41)	25,274 (0.21)	15,954 (0.23)
Hotel and restaurant	3,873 (0.03)	856 (0.01)	2,994 (0.03)	1,543 (0.02)
Information/communication	5,087 (0.04)	1,432 (0.02)	4,126 (0.03)	2,228 (0.03)
Manufacturing	14,306 (0.12)	7,560 (0.11)	13,694 (0.11)	7,429 (0.11)
Mining/quarrying	2,584 (0.02)	1,075 (0.02)	2,331 (0.02)	1,394 (0.02)
Other service	2,166 (0.02)	554 (0.01)	1,731 (0.01)	1,007 (0.01)
Public administration	3,765 (0.03)	5,528 (0.08)	6,850 (0.06)	3,756 (0.05)
Real estate	1,116 (0.01)	90 (0.00)	768 (0.01)	493 (0.01)
Transport and storage	10,150 (0.09)	3,292 (0.05)	8,687 (0.07)	4,952 (0.07)
Unknown or private households	91 (0.00)	5 (0.00)	61 (0.00)	50 (0.00)
Water supply	982 (0.01)	222 (0.00)	772 (0.01)	560 (0.01)
Wholesale and retail	24,783 (0.21)	3,132 (0.05)	17,763 (0.15)	10,964 (0.16)
**Company size**				
1 – 9	31,575 (0.26)	3,233 (0.05)	22,050 (0.18)	12,281 (0.18)
10 – 49	48,126 (0.40)	20,514 (0.30)	43,030 (0.36)	24,979 (0.36)
50 – 249	12,555 (0.11)	19,437 (0.28)	20,198 (0.17)	11,599 (0.17)

For peer review only - http://bmjopen.bmj.com/site/about/guidelines.xhtml

For peer review only

≥ 250	27,107 (0.23)	25,383 (0.37)	33,142 (0.28)	19,934 (0.29)
**Region**				
East	55,898 (0.47)	29,625 (0.43)	55,702 (0.47)	31,407 (0.46)
Middle	10,695 (0.09)	6,577 (0.10)	10,405 (0.09)	6,180 (0.09)
North	14,471 (0.12)	7,700 (0.11)	14,002 (0.12)	8,849 (0.13)
South	9,459 (0.08)	6,399 (0.09)	9,773 (0.08)	5,692 (0.08)
West	28,840 (0.24)	18,266 (0.27)	28,538 (0.24)	16,665 (0.24)
**Diagnosis**				
Musculoskeletal	43,419 (0.36)	21,570 (0.31)	40,752 (0.34)	24,199 (0.35)
Other types	53,417 (0.45)	34,490 (0.50)	55,200 (0.46)	32,385 (0.47)
Psychological	22,527 (0.19)	12,507 (0.18)	22,467 (0.19)	12,209 (0.18)

First, we fitted a multi-state model without covariates, to calculate the observed state probabilities in the entire cohort, regardless of IA status. A stacked probability plot is shown in Figure 2. All individuals started in full-time SA and could thereafter move to other states. The proportion of individuals who had returned to work reached 50.6 % (95% confidence interval (CI): 50.4 – 50.8) after 50 days, and 64.7 % (64.5 – 64.9) after 100 days. Just before passing one year, 77.0 % (76.8 – 77.2) were in work, 3.3 % (3.2 – 3.4) were in graded SA and 9.9 % (9.8 – 10.0) in full-time SA. At the same time, 9.2 % (9.0 – 9.3) were in non-employment and 0.5 % (0.47 – 0.53) in education. After one year we see distinct shifts in the probabilities, coinciding with maximum allowed days of consecutive sick leave benefit. After this, individuals either return to work or typically receive other welfare benefits, including work assessment allowance.

Main analysis

To assess the effect of IA versus no IA, we compared state probabilities in an IPT weighted multi-state model. A summary of individual characteristics in the weighted population can be found in Table 1. Figure 3 shows the absolute difference in state probabilities for IA versus no IA over the follow-up period, with 95% CI's based on 1000 bootstrap samples. In the figures, death was left out, as differences here were negligible. The figure shows how IA increased the probability of RTW and decreased the probability of full-time SA in the period following an initial SA episode. Peak effect of IA on RTW occurred after one month, where the probability was about 3.4 percentage points (pp) higher with IA.

The difference declined to below 2 pp after one year. The effect on full-time SA followed an opposite pattern for the first 2- 3 months and declined to zero after one year. The effect on graded SA was small (increase), but the confidence intervals included zero. The effect on non-employment was small initially but amounted to around 1 pp reduction after one year.

The average individual effects of IA versus no IA are summarized in terms of ELOS in Table 2. Here, we see that the estimated ELOS difference between IA and no IA for the first year was 8.4 more days (95% CI: 4.9 – 11.9 days) in work, 7.6 (4.8 – 10.3) fewer days in full-time SA and 1.6 (-0.2 – 3.4) fewer days in non-employment. Differences for graded SA and education were smaller.

Table 2 Expected length of stay (ELOS) in days, for one year after entering long-term full-time sickness absence (SA) from work, with 95% confidence intervals, based on 1000 bootstrap samples. The last column shows the difference in ELOS between IA and no IA, where an asterisk indicates confidence intervals of the difference that do not include zero.

	IA	No IA	Difference
**Full study population**			
Full-time SA	88.8 (87.2 – 90.5)	96.4 (95.3 – 97.5)	-7.6 (-10.3 – -4.8) *
Graded SA	18.1 (17.2 – 19)	17.3 (16.7 – 17.9)	0.8 (-0.7 – 2.2)
Work	240.8 (238.7 – 242.8)	232.4 (230.9 – 233.8)	8.4 (4.9 – 11.9) *
Non-employment	16.2 (15.1 – 17.3)	17.8 (17.1 – 18.5)	-1.6 (-3.4 – 0.2)
Education	0.9 (0.7 – 1.1)	1.0 (0.8 – 1.1)	-0.1 (-0.5 – 0.3)
**Women**			
Full-time SA	94.2 (92.3 – 96.1)	100.0 (98.3 – 101.7)	-5.8 (-9.4 – -2.2) *
Graded SA	21.9 (20.7 – 23.2)	21.9 (21.1 – 22.8)	0.0 (-2.1 – 2.1)

For peer review only

For peer review only - http://bmjopen.bmj.com/site/about/guidelines.xhtml

Work	232.3 (230.0 – 234.6)	226.3 (224.2 – 228.4)	6.0 (1.6 – 10.4) *
Non-employment	15.3 (14.0 – 16.6)	15.5 (14.4 – 16.5)	-0.2 (-2.5 – 2.1)
Education	1.2 (0.8 – 1.6)	1.2 (1.0 – 1.5)	0.0 (-0.6 – 0.6)
**Men**			
Full-time SA	82.9 (80.3 – 85.5)	92.3 (91.0 – 93.6)	-9.4 (-13.3 – -5.5) *
Graded SA	13.4 (12.2 – 14.7)	12.0 (11.4 – 12.6)	1.4 (-0.4 – 3.2)
Work	250.3 (247.2 – 253.5)	239.1 (237.4 – 240.8)	11.2 (6.4 – 16.1) *
Non-employment	17.5 (15.6 – 19.3)	20.6 (19.8 – 21.4)	-3.1 (-5.8 – -0.5) *
Education	0.6 (0.3 – 0.8)	0.7 (0.6 – 0.9)	-0.1 (-0.6 – 0.3)
**Musculoskeletal**			
Full-time SA	92.0 (89.2 – 94.8)	100.3 (98.4 – 102.1)	-8.3 (-12.9 – -3.7) *
Graded SA	20.2 (18.7 – 21.8)	18.7 (17.9 – 19.6)	1.5 (-1.0 – 3.9)
Work	236.0 (232.4 – 239.6)	227.5 (225.2 – 229.8)	8.5 (2.6 – 14.4) *
Non-employment	16.1 (14.3 – 17.8)	17.7 (16.7 – 18.6)	-1.6 (-4.3 – 1.1)
Education	0.7 (0.4 – 1.1)	0.8 (0.5 – 1)	0.0 (-0.6 – 0.5)
**Psychological**			
Full-time SA	104.3 (100.1 – 108.6)	115.4 (112.7 – 118.1)	-11.1 (-18.0 – -4.2) *
Graded SA	22.4 (20.2 – 24.5)	21.2 (19.9 – 22.5)	1.2 (-2.2 – 4.6)
Work	213.0 (207.9 – 218.1)	198.9 (195.9 – 201.9)	14.1 (6 – 22.3) *
Non-employment	23.9 (20.7 – 27.1)	27.7 (26.0 – 29.3)	-3.7 (-8.6 – 1.2)
Education	1.1 (0.6 – 1.7)	1.7 (1.3 – 2.2)	-0.6 (-1.6 – 0.4)

Subgroup analyses

Results from the subgroup analyses are plotted in Figure 3. Similar effect patterns as before were found, however, effects of IA were higher for men than for women. Effects were also higher in both selected diagnosis groups compared to in the overall analysis, although only slightly for musculoskeletal diagnoses.

For women, the effect of IA on RTW from full-time SA, reached a peak after 25 days, where the probability was 2.8 pp (95%CI: 1.9 – 3.7) higher with IA. The effect remained close to 2 pp higher for roughly 200 days, before declining to around 1 pp one year after inclusion. After one year, the effect was close to zero. The effect on work was almost entirely offset by reduced probability of full-time SA, while effects on other states were smaller. The peak effect for men on RTW were 29 days after starting full-time SA, with 4.5 pp (3.0 – 6.0) higher probability for men with IA. Contrary to in women, the effect on work stayed positive, never dropping below 2 pp, the entire follow-up period. The effect on work was mostly offset by reduced probability of full-time SA, but with more noticeable effects on other states, compared to women. There was a small increase in graded SA (after initially starting in full-time). Furthermore, there was a clear reduction in non-employment of about 2 pp after one year for men with IA at the time the initial SA started.

Workers with musculoskeletal illnesses as cause for their initial SA, reached peak effect of IA on RTW after 27 days, where the probability was 3.7 pp (2.4 – 5.0) higher than without IA. This effect magnitude was relatively stable until around day 100, from where it declined and then fluctuated around 2 pp to the end of follow-up. The effect on work was mostly offset by reduced probability of SA, though a slight increase for graded SA was seen and a 2 pp lower probability of non-employment after one year.

In the group with psychological diagnoses, the effect on RTW reached a maximum after 37 days, with 5.6 pp (3.5 – 7.7) higher probability with IA. Compared to other subgroups, effects varied much more over time. From the maximum effect of 5.6 pp, it dropped down to a 3 pp after one month, before increasing to over 5 pp three months later. The effect then varied between 3 and 5 pp before dropping down to 2 pp after one year. In the first year, the effect on RTW was mostly offset by reduced full-time SA, but a small increase in graded SA and reduction in non-employment were seen. One year after an initial SA episode due to psychological illnesses, the probability of non-employment was more than 2 pp lower with IA.

For peer review only - http://bmjopen.bmj.com/site/about/guidelines.xhtml

Average effects of IA versus no IA for subgroups are summarized in Table 2 in terms of ELOS over the first year. For women, we found that IA led to on average 5.8 (95% CI: 2.2 – 9.4) fewer days in full-time SA and 6.0 (1.6 – 10.4) more days in work. ELOS in other states were not affected. In men, effects were larger. Men with IA could expect 9.4 (5.5 – 13.3) fewer days in full-time SA and 11.2 (6.4 – 16.1) more days in work. There was a small increase in ELOS for graded SA of 1.4 days (-0.4 – 3.2), but the confidence interval included zero. Non-employment was lowered by 3.1 days (-5.8 – -0.5). For individuals with initial SA due to musculoskeletal illnesses, IA led to on average 8.3 (3.7 – 12.9) fewer days in full-time SA and 8.9 (3.3 – 14.5) more days in work. ELOS differences for graded SA and non-employment were small, and both confidence intervals included zero. Individuals with psychological diagnoses had the largest differences in ELOS. IA led to on average 14.1 (6.0 – 22.3) more days in work over the first year, and 11.1 (4.2 – 18.0) fewer days of full-time SA. Non-employment was reduced by 3.7 days for the psychological diagnosis group, but the confidence interval included zero.

Sensitivity analyses

To study the impact of covariate adjustment and to investigate robustness of our results, we conducted a series of sensitivity analyses, as described earlier. The analyses supported our main findings, but also indicated effect differences between industries. In the sensitivity analysis of the woman in the study population, where all diagnoses related to pregnancy, childbearing and family planning were removed, we found a somewhat higher effect of IA than in the analysis which included all the women. Detailed results from these analyses are described in sections S.2 to S.6 in the supplementary material and shown in Supplementary Figures 1-5.

The sequence plots that illustrate individuals' multi-state histories (see Supplementary Figure 6) showed that the most common history was one with a short period of full-time SA, less than 2 months, followed by uninterrupted stay in the work state. We have provided more details on the sequence plots in supplementary section S.7

A barplot that shows gender representation in the largest industries is included in Supplementary Figure 7 and further described in supplementary section S.8. Here we found that men worked mostly within construction, manufacturing and transport and women worked in education and health industries.

Discussion

We found that having access to the Norwegian IA Agreement, which included measures for preventing and reducing SA, at the time of initiating a first long-term SA episode, increased the probability of later work and decreased the probability of later full-time SA during the first 500 days after first entering SA. The probability of later non-employment was also reduced, particularly after 1 year. Larger effects were found for men than for women, and among individuals with musculoskeletal or psychological diagnoses connected to the initial SA spell. For women, the effect of IA was negligible after one year, while for men, the effect remained also after the first year.

A reduction in full-time SA and a slight increase in graded SA are in accordance with the first IA goal of reducing SA. Additionally, it is worth noticing that non-employment one year after entering SA, appears to be reduced in workplaces with IA, especially for men. From a health perspective, the underlying intention of IA is to reduce SA by remaining in work, also after the first year when the right to certain SA benefits in Norway ceases. Our results show that having IA contributes to reducing SA by keeping people in work, rather than by exiting work. Furthermore, while previous studies found that IA enterprises may have higher SA, or that IA has none or minor effects on the occurrence of SA, [8, 11, 12] our study shows that once on SA, IA contributed towards reducing the number of SA days. This could indicate that IA had a larger effect on SA duration than on the occurrence of SA.

A recent paper on the effect of IA on SA prevalence and duration, using similar data sources but other methods, found a beneficial effect of IA in terms of reduced duration for both musculoskeletal and psychological diagnoses, particularly in men. [12] Another study, from 2020, on IA and risk of long-term SA spells found small differences, but a slight decrease for female workers in stratified analyses. [11] Effects of IA are greater among individuals who had musculoskeletal or psychological diagnoses, compared to other diagnoses, is expected, as the intervention is specifically aimed at these patient groups.[6]

We estimated effects of having access to IA and did not investigate which IA measures had most effect or whether workers had accessed them. There is supporting evidence that various workplace health-promotion

For peer review only - http://bmjopen.bmj.com/site/about/guidelines.xhtml

interventions may prolong working life in certain types of work [25]. A Cochrane review from 2015 found moderate evidence for workplace interventions being effective in increasing RTW in workers on sick leave with musculoskeletal disorders (first and lasting RTW) and mental health problems (only first RTW) [26]. A systematic review from 2018 [27] further supports these findings. However, a review of studies on the effect of interventions for improving RTW in workers with common mental illness found that the available interventions did not lead to improved RTW rates [28]. One should also note, as IA has been gradually introduced at a national level in Norway over the study period and beyond, that it is likely that IA has had an impact on SA and RTW at a societal level. This effect is not picked up in our study, as we consider individual differences of having IA or not over the study period.

A strength of this study is the use of a large cohort with high completeness, with detailed information on individual longitudinal work-participation and a large set of confounders. Another strength is the use of multi-state modelling. We believe that the study serves as a good example of how longitudinal data from multiple registries can be used to construct detailed outcome trajectories and that IPT weighted multi-state models offer a suitable way of estimating effects on such outcomes. The approach allows us to analyse more detailed long-term work-related outcomes than traditional approaches, and to calculate various relevant outcome measures, ELOS being one example. However, there are also various limitations. Most importantly, this is an observational study, and access to the measures given through IA was not randomized, but subject to the decision of the company signing up for IA. Even though adjustment for various assumed confounding variables was made, there is no guarantee that residual confounding is not present. However, sensitivity analyses were not able to detect residual confounding in settings where more detailed confounder information was available. The negative control by analysing pre-exposure multi-state histories indicated the same. Note that if the negative control had showed different pre-exposure histories, even after initial confounder adjustment, these histories could also be adjusted for as additional baseline information.

Given the assumptions of no unmeasured confounding, positivity and consistency, the IPT weighted multi-state model aims to mimic the scenario one would see if access to IA was randomized at baseline (time of first long-term SA episode). Note also that we implicitly assume that IA exposure acts as if given at first SA. As our analyses are restricted to individuals without any recent SA history, we believe this to be a reasonable assumption. The mechanisms through which IA potentially promotes faster RTW are many. One can assume that some are connected to individual measures undertaken at IA workplaces, while others are related to measures available for IA workplaces in general.

There were notable differences in the estimated effects of IA for women and men, which can have various explanations. The Norwegian labour market has a high level of gender segregation [29, 30], which can also be observed in our study population. For our study sample, a high proportion of men worked in construction, manufacturing and transport, while women dominated the educational and healthcare industries. Although the available measures are the same for every IA company, it is reasonable to expect that the implementation and effect of IA measures vary substantially across workplaces, occupations and industries. The governmental appointed Research Group for the IA Agreement writes in their report from 2018 [31] that there are substantial differences between industries and types of work regarding challenges with SA and SA reducing measures. Also, as observed in most western countries over many years [32 - 34], women generally have a higher prevalence of SA than men. The gender differences in labour outcomes are the subject of extensive research and debate. Some studies have indicated that differences in work-related outcomes can be caused by unfavourable working conditions among women [35, 36], while others find less favourable conditions among men [33]. Some studies suggest women handle work-related strain worse than men [37]. There are also results indicating that the double burden of household work affects women to a higher degree than men [38], and if the SA comes as a result of challenges at home, measures focusing on the workplace might not be as effective. A qualitative study of interventions and rehabilitation activities in connection with RTW from 2021 [39] found that women expressed a need for home-related interventions, whereas men expressed a need for organizational interventions to counter feelings of resignation at work. As the IA Agreement is focused on workplace-related measures, it may therefore be the case that the IA measures better target men. Furthermore, a substantial part of the initial SA cases among women in our study population was in connection with pregnancy, childbearing and family planning, and IA measures may not be as relevant for SA tied to such diagnoses. Supplementary analysis showed that when excluding these cases, the effects of IA among the remaining women were somewhat higher.

The IA Agreement is specific to Norway, and it is not likely that similar effects would have been seen in other countries. Norway is known for having a generous welfare system and high protection for individual workers. Additionally, the cohort studied was young (28-43 years), and results do not necessarily generalise to older populations. Future work should focus on studying specific measures for preventing and reducing SA and how they vary with different types of work, industries, diagnosis types and other worker characteristics.

For peer review only - http://bmjopen.bmj.com/site/about/guidelines.xhtml

Contributorship statement

RH, NM and JMG conceptualised the study. NM, RaH, SLM, KU, PK, ISM and JMG contributed to the interpretation of the data and study design. RH and NM performed the data preparation and data analysis with the contribution of JMG. All authors contributed to the interpretation of the results. RH prepared the first draft of the manuscript. All authors commented on previous versions, contributed to revised versions and approved the final manuscript. The corresponding author attests that all listed authors meet authorship criteria and that no others meeting the criteria have been omitted.

Competing interests

None declared.

Funding

This work was supported by the Research Council of Norway (Grant number 273 674).

Data sharing statement

No data are available. The data underlying this article are considered sensitive and subject to strict regulation and cannot be shared publicly due to privacy of individuals included in the study.

R code for preparing individual multi-state histories and performing multi-state models is available on GitHub, see https://github.com/runhoff/IA/.

Ethics statements

Ethics approval and consent to participate. This research study was conducted retrospectively from data provided by third party national Norwegian registries and the use of the data was sanctioned under Regional Committees for Medical and Health Research Ethics (REC) license number 17 344. Informed consent is not needed for this registry-based study according to Norwegian law (The Personal Data Act, Section 8 and 9, and The General Data Protection Regulation Article 6.1 e) and Article. 9.2 j)).

Consent for publication. Not applicable.

Acknowledgments

The late Professor Emeritus Tor Bjerkedal (1926–2015) conceived the idea for and took part in establishing this registry-based cohort in 2002.

References

[1] Henderson M, Glozier N, Elliott K H. Long term sickness absence. *BMJ* 2005;330:802–803.

[2] Waddell G, Burton A K. Is Work Good for Your Health and Well-being? The Stationery Office, London 2006.

[3] Gustavsson A, Bjorkman J, Ljungcrantz C, Rhodin A, Rivano-Fischer M, Sjolund KF, Mannheimer C. Socio-economic burden of patients with a diagnosis related to chronic pain–Register data of 840,000 S wedish patients. *Eur J Pain* 2012;16(2):289-99.

[4] Kivimäki M, Ferrie J E, Hagberg J, et al. Diagnosis-specific sick leave as a risk marker for disability pension in a Swedish population. *J Epidemiol Community Health* 2007;61(10):915-20.

For peer review only - http://bmjopen.bmj.com/site/about/guidelines.xhtml
For peer review only

[5] Karlsson N E, Carstensen J M, Gjesdal S, et al. Risk factors for disability pension in a population-based cohort of men and women on long-term sick leave in Sweden. *Eur. J. Public Health* 2008;18(3):224–231.

[6] Arbeids- og sosialdepartementet: Intensjonsavtale om et inkluderende arbeidsliv - budsjettmessige konsekvenser mv. (2001). https://www. regjeringen.no/no/dokumenter/stprp-nr-1-tillegg-nr-1-2001-2002-/ Accessed 2020-08-03.

[7] Gjesdal S. Sykefraværets utvikling i Norge 1975–2002. *Tidsskr. Nor. Lægeforen.* 2005;125:742–5.

[8] Lie A. "Inclusive Working Life" in Norway" – Experience from "Models of Good Practice" *Enterprises. Croat. Med. J.* 2008;49(4):553–560.

[9] Ministry of Labour. Letter of Intent regarding a more inclusive working life 2006–2009 [2007]. https://www.regjeringen.no/globalassets/ upload/aid/temadokumenter/velferd/ia/a more inclusive working life 2006-2009.pdf Accessed 2021-10-04.

[10] Foss L, Gravseth H M, Kristensen P, et al. "Inclusive working life in Norway": a registry-based five-year follow-up study. *J. Occup. Med. Toxicol*. 2013;8(1):1–8.

[11] Hanvold T N, Kristensen P, Corbett, K, et al. Long-term sickness absence among young and middle-aged workers in Norway: the impact of a population-level intervention. *BMC Public Health* 2020:20(1);1–12.

[12] Hasting R L, Merkus S L, Hanvold T N, et al. Impact of the Norwegian Agreement for a More Inclusive Working Life on diagnosis-specific sickness absence in young adults: a difference-in-difference analysis. *BMC Public Health* 2022 ;22(1):1-13.

[13] Odeen M, Magnussen L H, Mæland S, et al. Systematic review of active workplace interventions to reduce sickness absence. *Occup. Med*. 2013;63(1):7–16.

[14] Steenstra I A, Anema J R, Van Tulder M W, et al. Economic evaluation of a multi-stage return to work program for workers on sick-leave due to low back pain. *J. Occup. Rehabil*. 2006;16(4):557–578.

[15] Andersen P K, Keiding N. Multi-state models for event history analysis. *Stat Methods Med Res.* 2002;11(2):91–115.

[16] Gran J M, Lie S A, Øyeflaten I, et al. Causal inference in multi-state models–sickness absence and work for 1145 participants after work rehabilitation. *BMC Public Health* 2015;15(1):1082.

[17] Kristensen P, Bjerkedal T. Educational attainment of 25 year old Norwegians according to birth order and gender. *Intelligence* 2010;38(1):123–136.

[18] Carré H. Statistical Classification of Economic Activities in the European Community. Publications Office of the European Union, Luxembourg 2008.

[19] Verbeke M, Schrans D, Deroose S, et al. The international classification of primary care (icpc-2): an essential tool in the EPR of the GP. *Stud. Health Technol. Inform*. 2006;124:809.

[20] Lipsitch M, Tchetgen E T, Cohen T. Negative controls: a tool for detecting confounding and bias in observational studies. *Epidemiolog*y 2010;21(3):383.

[21] Grand M K, Putter H. Regression models for expected length of stay. *Stat. Med.* 2016;35(7):1178–1192.

[22] Hernan M, Robins J M. Causal Inference: What If. Chapman & Hall/CRC, Boca Raton 2020.

[23] Aalen O O, Borgan Ø, Gjessing H. Survival and Event History Analysis: a Process Point of View. Springer, New York, NY 2008.

For peer review only - http://bmjopen.bmj.com/site/about/guidelines.xhtml

[24] Xiao Y, Abrahamowicz M. Bootstrap-based methods for estimating standard errors in Cox's regression analyses of clustered event times. *Stat. Med*. 2010;29(7-8):915–923.

[25] Nielsen R A, Midtsundstad T I. Do workplace health-promotion interventions targeting employees with poor health reduce sick-leave probability and disability rates? *Scand. J. Public Health* 2020;49(2):219–227

[26] van Vilsteren M, van Oostrom SH, de Vet HC, Franche RL, Boot CR, Anema JR. Workplace interventions to prevent work disability in workers on sick leave. *Cochrane Database Syst. Rev*. 2015(10).

[27] Cullen, K.L., Irvin, E., Collie, A. *et al.* Effectiveness of Workplace Interventions in Return-to-Work for Musculoskeletal, Pain-Related and Mental Health Conditions: An Update of the Evidence and Messages for Practitioners. *J Occup Rehabil.* **28**, 1–15 (2018). https://doi.org/10.1007/s10926-016-9690-x

[28] Nigatu YT, Liu Y, Uppal M, McKinney S, Rao S, Gillis K, et al. Interventions for enhancing return to work in individuals with a common mental illness: systematic review and meta-analysis of randomized controlled trials. *Psychol. Med*. 2016;46(16):3263–74.

[29] Ellingsæter A L. Scandinavian welfare states and gender (de) segregation: Recent trends and processes. *Econ. Ind. Democr*. 2013;34(3):501-518.

[30] Ose S O, Jiang L, Bungu B. Det kjønnsdelte arbeidsmarkedet og kvinners arbeidshelse [The gender-segregated labor market and women's occupational health]; 8214056780; SINTEF: 2014.

[31] Research Group for the IA Agreement. Målene om et mer inkluderende arbeidsliv - status and utviklingstrekk [goals for a more inclusive working life - status and developments]. Report 2017. Oslo: Arbeids- og sosialdepartementet; 2018. Accessed 25 Aug 2022. [Available from: https://www.regjeringen.no/globalassets/departementene/aid/dokumenter/2018/ia-rapport_2018_web.pdf]

[32] Mastekaasa A. The gender gap in sickness absence: long-term trends in eight European countries. *Eur. J. Public Health* 2014;24(4):656-62.

[33] Mastekaasa A, Melsom AM. Occupational segregation and gender differences in sickness absence: evidence from 17 European countries. *Eur. Sociol. Rev*. 2014;30(5):582-94

[34] Gajewski K, Burris D, Spears DR, Sullivan K, Oyinloye O, McNeil C, Meechan P, Warnock E, Trapp J, Decker KC, Chapman S. Demographic trends of sick leave absenteeism among civil service employees at a federal agency from 2004 to 2012. *J. Occup. Environ*. 2015;57(3):277-83.

[35] Laaksonen M, Mastekaasa A, Martikainen P, Rahkonen O, Piha K, Lahelma E. Gender differences in sickness absence-the contribution of occupation and workplace. *Scand J Work Environ Health* 2010:394-403.

[36] Labriola M, Holte KA, Christensen KB, Feveile H, Alexanderson K, Lund T. The attribution of work environment in explaining gender differences in long-term sickness absence: results from the prospective DREAM study. *Occup. Environ. Med*. 2011;68(9):703-5.

[37] Hooftman WE, Van Der Beek AJ, Bongers PM, Van Mechelen W. Is there a gender difference in the effect of work-related physical and psychosocial risk factors on musculoskeletal symptoms and related sickness absence?. *Scand J Work Environ Health*. 2009:85-95.

[38] Voss M, Josephson M, Stark S, Vaez M, Alexanderson K, Alfredsson L, Vingård E. The influence of household work and of having children on sickness absence among publicly employed women in Sweden. *Scand J Public Health* 2008;36(6):564-72.

[39] Nybergh L, Bergström G, Jensen I, Hellman T. Experiences of interventions and rehabilitation activities in connection with return-to-work from a gender perspective. A focus group study among employees on sick leave for common mental disorders. *PLoS One* 2021;16(6):e0253049.

For peer review only - http://bmjopen.bmj.com/site/about/guidelines.xhtml

Figures

Figure 1 The multi-state model showing possible transitions after individuals start in in full-time sickness absence (SA). Individuals may move repeatedly between full-time SA, graded SA, work, non-employment and education. Note that death was a competing transition in all states, but left out of the figure

Figure 2 Stacked probability plot of estimated unadjusted state probabilities, for the full study population (both intervention groups, n = 187 930), after first initial entry into full-time sickness absence (SA). Individuals may have repeated spells of each state during follow-up, and the plot shows the probability of being in either of the states at all days during the follow-up period.

Figure 3 Difference in estimated state probabilities, $\pi^1(t)-\pi^0(t)$, for 500 days following initial entry into full-time sickness absence (SA), where superscript 1 denotes having the Inclusive Working Life Agreement (IA) group and superscript 0 denotes not having IA. Estimates were adjusted for confounding using inverse probability of treatment (IPT)weighting. Faded areas are 95 % confidence intervals based on 1000 bootstrap samples. The topmost plot in the figure shows results for the entire study population. The middle two plots are for women and men separately, and the bottom two are separated between musculoskeletal and psychological diagnoses pertaining to the initial SA spell.

For peer review only

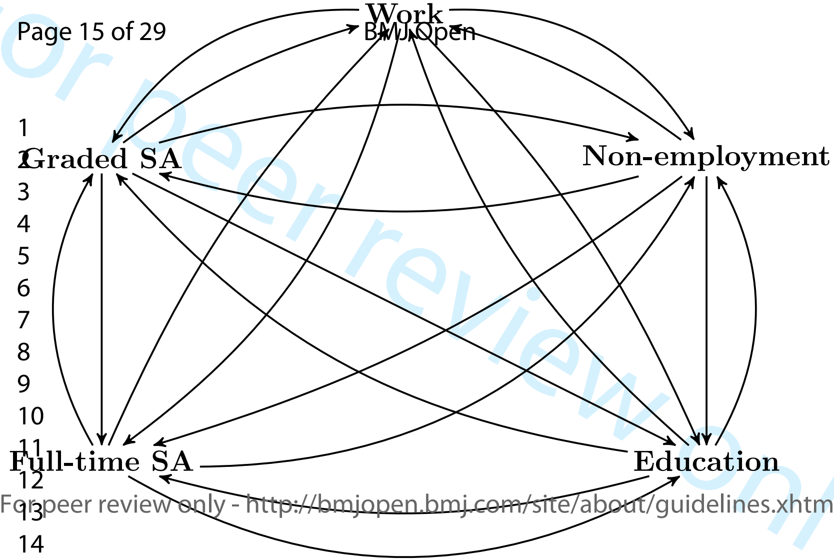
Work
Graded SA
Non-employment
Full-time SA
Education

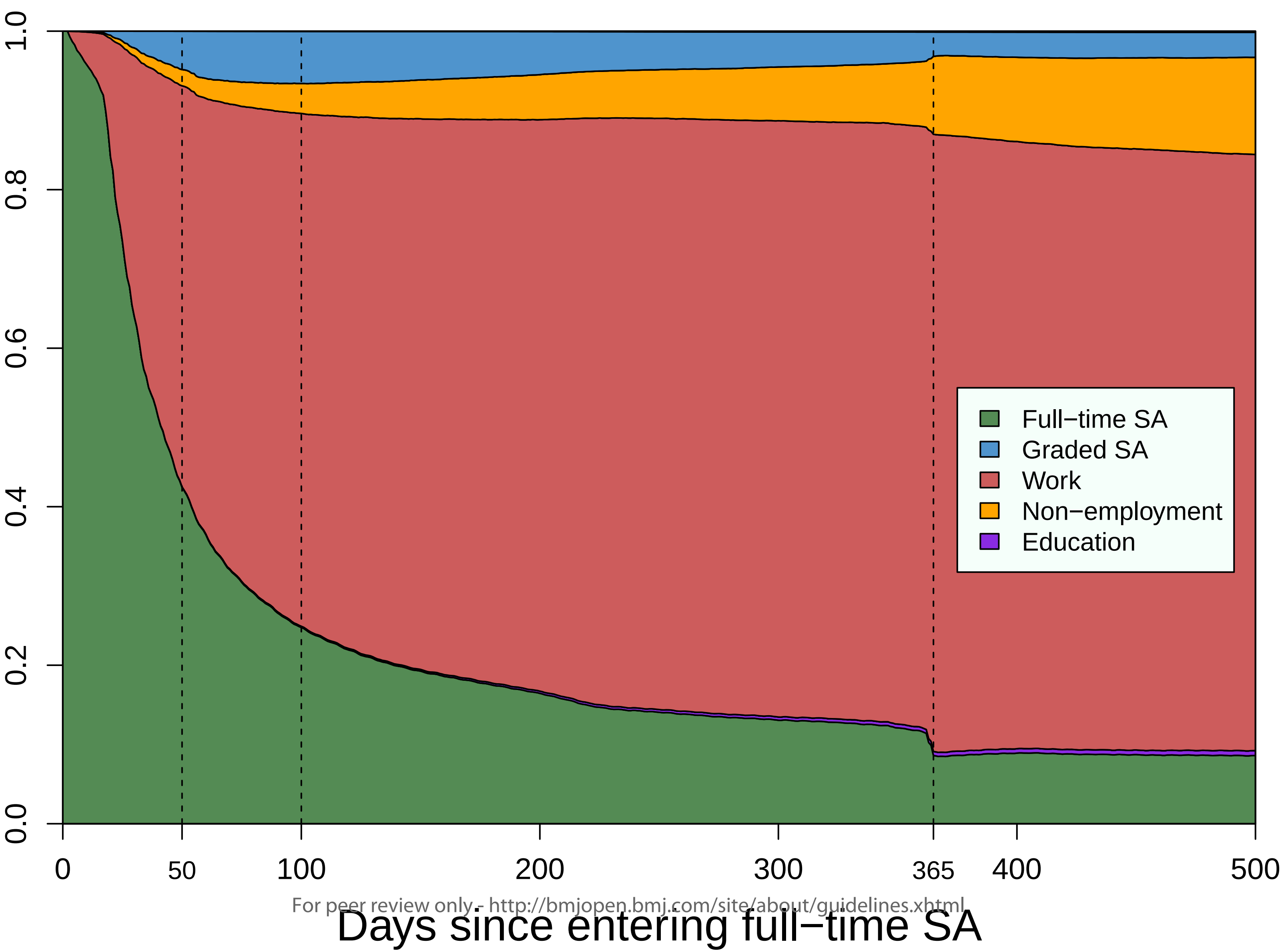

For peer review only - http://bmjopen.bmj.com/site/about/guidelines.xhtml

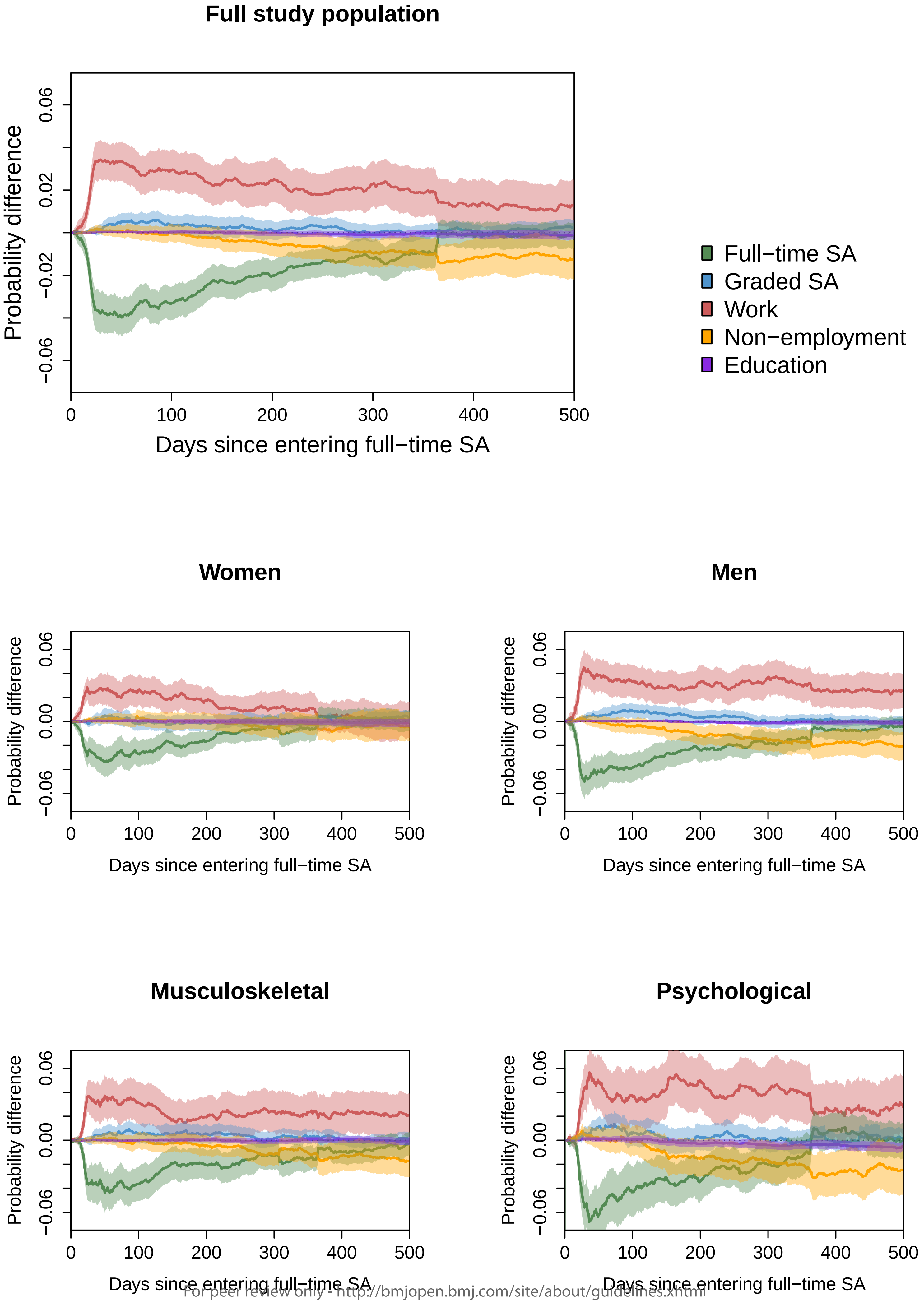
Full study population
Probability difference
0.06
0.02
−0.02
−0.06
0
100
200
300
400
500
Days since entering full−time SA
Full−time SA
Graded SA
Work
Non−employment
Education
Women
Men
0.00
Musculoskeletal
Psychological

An initiative for a more inclusive working life and its effect on return-to-work after sickness absence – a multi-state longitudinal cohort study

Supplementary Material

S.1 Estimands and statistical methods

A multi-state model can be seen as a stochastic process $\{X(t) \mid t \geq 0\}$, taking values from a set of possible states S = {1, 2, 3, ..., S} [1, 2, 3]. In the present study, S = 6, and *t* was days since inclusion. The model is governed by transition intensities $\alpha_{jk}(t)$, referring to the probability of a jump to state *k* from state *j* within a small time interval Δt, divided by Δt, as Δt approaches 0:

$$\alpha_{jk}(t) = \lim_{\Delta t \to 0} \frac{\Pr(X(t + \Delta t) = k \mid X(t-) = j)}{\Delta t}$$

for all j,k in S, where j ≠ k.

When $\boldsymbol{A(t)}$ is the matrix of cumulative transition intensities with elements $A_{jk}(t) = \int_0^t \alpha_{jk}(s)\, ds$, for $j \neq k$ and $A_{jj}(t) = -\sum_{j \neq k} A_{jk}(t)$, the state probabilities $\boldsymbol{\pi}(t)$ with elements $\pi_j(t) = \Pr(X(t) = j)$ are found using the Aalen-Johansen estimator [4], by

$$\widehat{\boldsymbol{\pi}}(t) = \boldsymbol{\pi}(0) \prod_{(0,t]} (\mathbf{I} + \Delta \boldsymbol{A}(u)) \tag{1}$$

where $\boldsymbol{\pi}(0)$ is the initial state distribution at time zero. In settings with time-to-event data, right censoring and no covariates, the elements of $\boldsymbol{A}(t)$ can be estimated using the Nelson-Aalen estimator [4]. State probabilities (also known as state occupational probabilities) are a simple and intuitive quantities for multi-state outcomes and can be seen as an extension of regular survival probabilities (e.g., from Kaplan-Meier curves).

To assess the effect of the Norwegian Inclusive Working Life Agreement (IA), and the within measures for preventing and reducing SA, we sought to identify the average treatment effect (ATE), as the difference in state probabilities

$$\theta(t) = \pi_j^1(t) - \pi_j^0(t) \tag{2}$$

for all j ∈ S, where $\boldsymbol{\pi}^a(t)$ are the counterfactual state probabilities if everyone in the study population was fixed to IA status *a* (taking the values 1 and 0 for IA and non-IA respectively).

From the state probabilities we calculated

$$\theta = \int_0^{365} \pi_j^1(t)\, dt - \int_0^{365} \pi_j^0(t)\, dt \tag{3}$$

representing the difference in expected length of stay (ELOS) in state j, over the first year, for the situations where everyone was fixed to $a = 1$ and $a = 0$.

For peer review only - http://bmjopen.bmj.com/site/about/guidelines.xhtml

The estimands in formula (2) and (3) can be identified under conditional exchangeability (no unmeasured confounding), positivity and consistency [5]. Adjustment for confounding was made by applying baseline inverse probability of treatment (IPT) weights [5], also known as propensity score weights, while fitting hazard models for every possible transition in the model.

More precisely, we fitted a weighted additive hazards regression model [4] on the form

$$\alpha_{jk}(t\,|a) = \beta_{jk0}(t) + \boldsymbol{\beta}_{jk}^{T}(t)a$$

for all possible transitions jk, where $\boldsymbol{\beta}^{T}$ (t) are time-varying regression functions and $\beta_{jk0}(t)$ is the baseline j → k transition intensity. For each observation we applied the baseline inverse probability of treatment (IPT) weight $\widehat{w}_i$, for the corresponding individual i. Estimates $\widehat{\boldsymbol{B}}_{jk}(t)$of the cumulative regression functions

$$\boldsymbol{B}_{jk}(t) = \left[\int_0^t \beta_{jk0}(u)du\,, \int_0^t \beta_{jk}^{T}(u)du\right]^{T}$$

are found using least squares techniques [4]. For the two possible values of a, we then found the cumulative transition intensity matrices by

$$\hat{A}_{jk}^{a}(t) = \hat{A}_{jk}(t|a) = \widehat{\boldsymbol{B}}_{jk}^{T}(t)\boldsymbol{a}^{*}$$

where $\boldsymbol{a}^{*} = (1, a)^{T}$, which again was plugged into the formula in (1), and thus identify the estimands in formula (2) and (3).

To calculate the IPT weights $\widehat{w}_i$, we modelled IA status A, given baseline confounders **Z** in a logistic regression model

$$logit\,\Pr(A|\,\mathbf{Z}) = \beta^{T}\mathbf{Z}$$

Using this model, for each individual i, the conditional probability of having their assigned IA status a_i given their covariate values z_i at baseline, $\Pr(a_i \mid \boldsymbol{z}_i)$ were estimated and used to calculate the (stabilized) IPT weight [5]

$$\widehat{w}_i = \frac{\Pr(a_i)}{\Pr(a_i\,|\boldsymbol{z}_i)}$$

where $\Pr(a_i)$ is the sample proportion with IA status equal to a_i at baseline.

S.2 Sensitivity analysis 1: Using subsets of adjustment variables

As a first sensitivity analysis, we compared the results from the main analysis with results from a series of analyses adjusting for all possible subsets of the main adjustment set, including the empty set – in total 256 models. Results are shown in Supplementary Figure 1.

In the left panel of Supplementary Figure 1, the unadjusted differences in state probabilities are seen to be almost twice as big as after confounder adjustment using IPT weighting. While in the right panel, we see how including smaller and less complete sets of confounders can lead to shifts in both directions compared to the main model.

For peer review only - http://bmjopen.bmj.com/site/about/guidelines.xhtml
For peer review only

S.3 Sensitivity analysis 2: Adjusting for additional covariates measured for men during military

Most of the men included in the study underwent military conscript examinations at the age of 18. Here they measured, among other things, IQ score, BMI, physical stamina score and a rough assessment of military eligibility (physical and mental). These data were available to us from the Norwegian Armed Forces Personnel Data Base. As these variables were not measured for all the individuals in the cohort, especially women, they were not adjusted for in the primary analyses. However, as a sensitivity analysis, we compared weighed analysis for men not taking and taking these variables into account. The results are shown in the left panel in Supplementary Figure 2. The plot shows no visible differences. In fact, the absolute differences in effect estimates came after the third – fourth decimal. We also tried adding conscript variables in an alternative analysis where the variables for education and workplace industry from the main analysis were left out. Here we were able to detect visible differences in effect estimates from the plot. Results of this analysis are shown in the right panel of Supplementary Figure 2.

S.4 Sensitivity analysis 3: Pre IA (1997-1999) multi-state histories as a negative control

Individual multi-state histories are also available for individuals in the cohort before IA was introduced and can be utilised as a negative outcome control [6]. The question of interest is then whether these earlier multi-state histories differed between those with later IA and those without, and if the IPT weights used sufficiently adjusted for such a difference. We therefore considered state histories during the period from 1st of January 1997 to 31st of December 1999, where we compared both the crude difference in state probabilities for the same intervention groups as in the main analysis, and a weighted analysis using the IPT weights of the main analysis. The results are found in Supplementary Figure 3. Results showed that the later IA groups differed between 1997 and 2000 in terms of education probability (IA group about 4 percentage points more likely to be in education) and work probability (non-IA group about 3 percentage points more likely to be in work). For non-employment there were only small differences. The probabilities for full-time or graded SA did not differ much, as one would expect due to the restriction of no history of SA one year before inclusion. However, after applying the IPT weights from the main analysis, also the differences in work histories that were there disappeared. This indicates that the confounding adjustment through these weights sufficiently covered differences in earlier work life (all states) histories.

S.5 Sensitivity analysis 4: Analysis of a limited selection of industries

From the information in Table 2 in the main article, it appeared that a large proportion of IA companies were in industries where public workplaces are typically overrepresented, such as education and health services. Information on public vs private workplace was not available for the study. In this section, we performed a separate analysis for a limited selection of industries, assumed to have many private workplaces, namely wholesale and retail, construction, manufacturing, commercial services and transport and storage. Results from these analyses are found in Supplementary Figure 4. We found a higher effect of IA within this subgroup, more akin to the effect seen in men in the main article.

S.6 Sensitivity analysis 5: Analysis with initial SA spells related to pregnancy, childbearing and family planning removed

Since our study population were in an age span 28-42, a high proportion of the initial SA cases were related to pregnancy, childbearing and family planning. We removed 20355 cases with such diagnosis types and ran the analysis for the remaining women. The results can be seen in Supplementary Figure 5. As can be seen in the Figure, there were an increased effect of IA among the remaining women after these diagnoses were removed.

For peer review only - http://bmjopen.bmj.com/site/about/guidelines.xhtml

Roughly one month after inclusion, we found that by removing these cases, the effect of IA on work was increased in the range of 0.4-0,6 pp higher probability of work, while being offset by similar reduction for being in SA. The effect difference is noticeable smaller and almost non-existent 100 days into the study and this pattern is seen for a good hundred days going forward. Towards the end of the study, we again see some increase in the effect on work in this limited stratum, but now the increased effect on work is offset by an even greater reduction in non-employment than seen in the full female population.

S.7 Sequence analysis of state trajectories within different industries

To illustrate how typical patterns of individual state trajectories differ between different industries and IA status, we made so-called sequence plots using the TraMineR package in R. Different industries with similar characteristics were grouped together. In order to plot individual trajectories with some form of clarity, we based the plots on 500 randomly sampled individuals within each stratum. The sequence plots are displayed in Supplementary Figure 6. Here, the state histories are sorted backwards after similar states, and indications of some differences in common patterns between the industries can be seen.

In all industries, the by far most common type of individual state history is that consisting of a shorter period of full-time SA, typically less than 2 months, followed by an uninterrupted stay in the work state. Furthermore, the increased amount of work seen in the IA intervention group, seems to come from individuals who are not in work at the final observation day, but rather have varying length of work periods in the middle of the observation time span. Partial SA appears to be less frequently used within construction and manufacturing industries.

S.8 Barplot showing number of individuals in the six biggest industries

The barplot in Supplementary Figure 7 illustrates the segregated labour market, where women typically work within health and education, while men are overrepresented in construction, manufacturing and transport industries.

References

[1] Hougaard P. Multi-state models: a review. Lifetime Data Analysis. 1999;5(3):239–264.

[2] Andersen PK, Keiding N. Multi-state models for event history analysis. Stat Methods Med Res. 2002;11(2):91–115.

[3] Putter H, Fiocco M, Geskus RB. Tutorial in biostatistics: competing risks and multi-state models. Stat Med. 2007;26(11):2389–2430.

[4] Aalen OO, Borgan Ø, Gjessing H. Survival and event history analysis: a process point of view. New York, NY: Springer; 2008.

[5] Hernan M, Robins JM. Causal Inference. Boca Raton: Chapman & Hall/CRC; 2020.

[6] Lipsitch M, Tchetgen ET, Cohen T. Negative controls: a tool for detecting confounding and bias in observational studies. Epidemiology (Cambridge, Mass). 2010;21(3):383.

[7] Gabadinho, A., G. Ritschard, N.S. Müller and M. Studer. Analyzing and Visualizing State Sequences in R with TraMineR. Journal of Statistical Software. 2011; 40(4).

For peer review only

Tables

Supplementary Table 1 Total number of transitions made by the study population of n = 187 930 individuals, over 90 231 405 person days, during individual follow-up of up to 500 days after entering full-time sickness absence (SA).

**FROM \ TO**	Full-time SA	Work	Graded SA	Non-employment	Education	Death
Full-time SA	0	197 774	32939	18760	492	163
Work	64 888	0	18562	37074	2003	78
Graded SA	9127	35254	0	1853	49	0
Non-employment	5902	28468	750	0	1015	34
Education	131	1526	31	737	0	0
Death	0	0	0	0	0	0

Figures

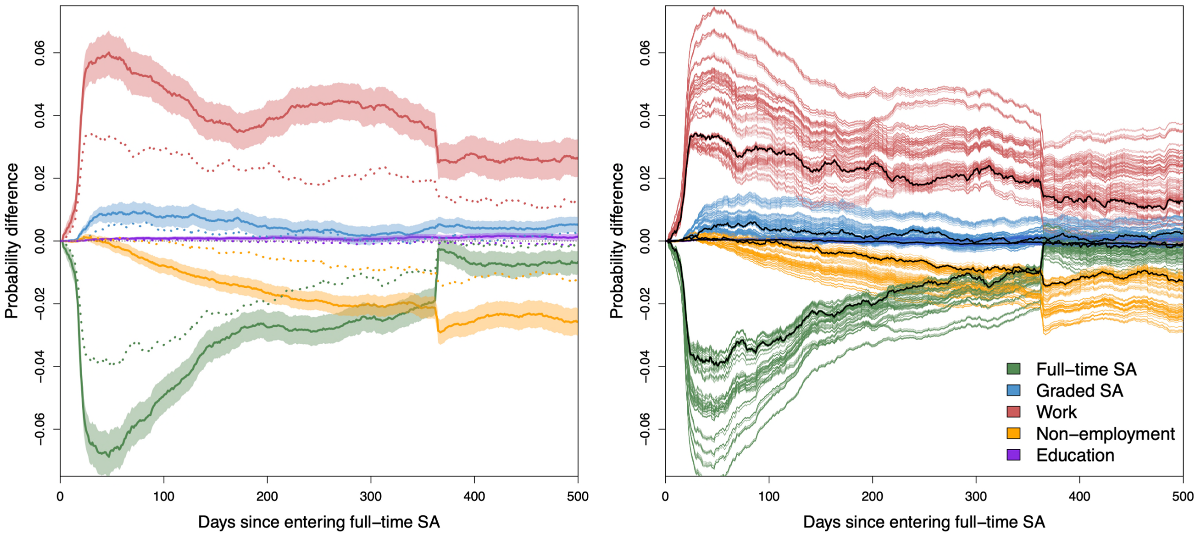

Supplementary Figure 1: Differences in estimated state probabilities, $\hat{\boldsymbol{\pi}}^1(t) - \hat{\boldsymbol{\pi}}^0(t)$, for the full study population, after entering long-term full-time sickness absence (SA), where superscript 1 denotes having the Inclusive Working Life Agreement (IA) and superscript 0 denotes not having IA. Faded areas are 95 % confidence intervals based on 1000 bootstrap samples. **Left:** The full-drawn lines are unadjusted estimates, while the dotted lines show the inverse probability of treatment (IPT) weighted estimates from the main article. **Right:** The black lines illustrate the IPT weighted estimates from the primary analysis in the main paper and colored lines are IPT weighted estimates from using different subsets of the main adjustment set when constructing the weights.

For peer review only - http://bmjopen.bmj.com/site/about/guidelines.xhtml

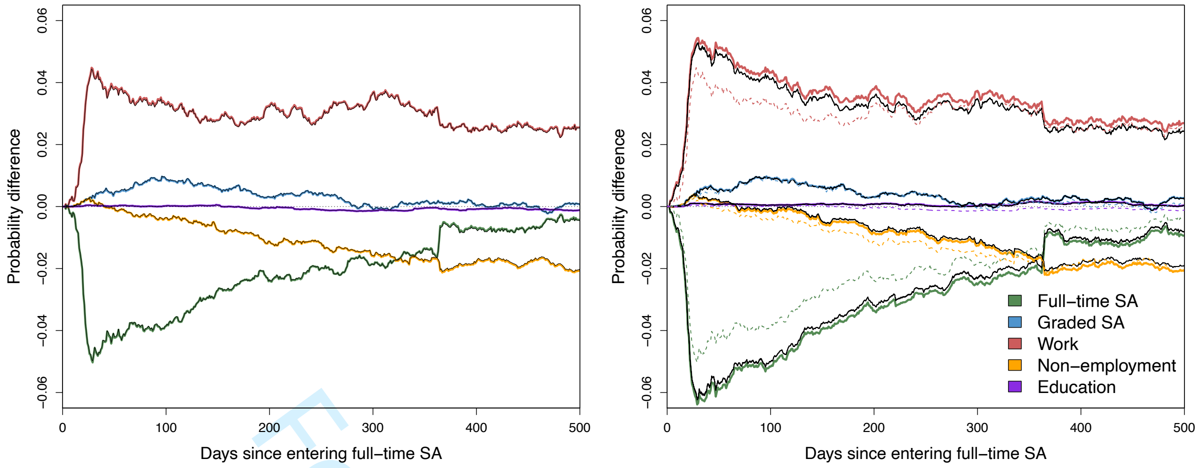

Supplementary Figure 2: Difference in estimated state probabilities, $\hat{\boldsymbol{\pi}}^1(t) - \hat{\boldsymbol{\pi}}^0(t)$, for men who completed military conscript examinations, after entering long-term full-time sickness absence (SA), where superscript 1 denotes having the Inclusive Working Life Agreement (IA) and superscript 0 denotes not having IA. **Left:** The colored lines are estimates adjusted for confounding by inverse probability of treatment (IPT) weighting using the main confounder set. Black lines show the estimates from when conscript variables IQ score, BMI, physical stamina test and military eligibility test were also adjusted for. **Right:** The black lines show estimates where also conscript variables IQ score, BMI, physical stamina test and military eligibility test were adjusted for but leaving out the covariates for education and workplace industry. Full drawn colored lines are estimates adjusted for confounding by IPT weighting, leaving out education and workplace industry from the confounder set. Dotted colored lines are original estimates adjusted for confounding using the full main adjustment set.

For peer review only

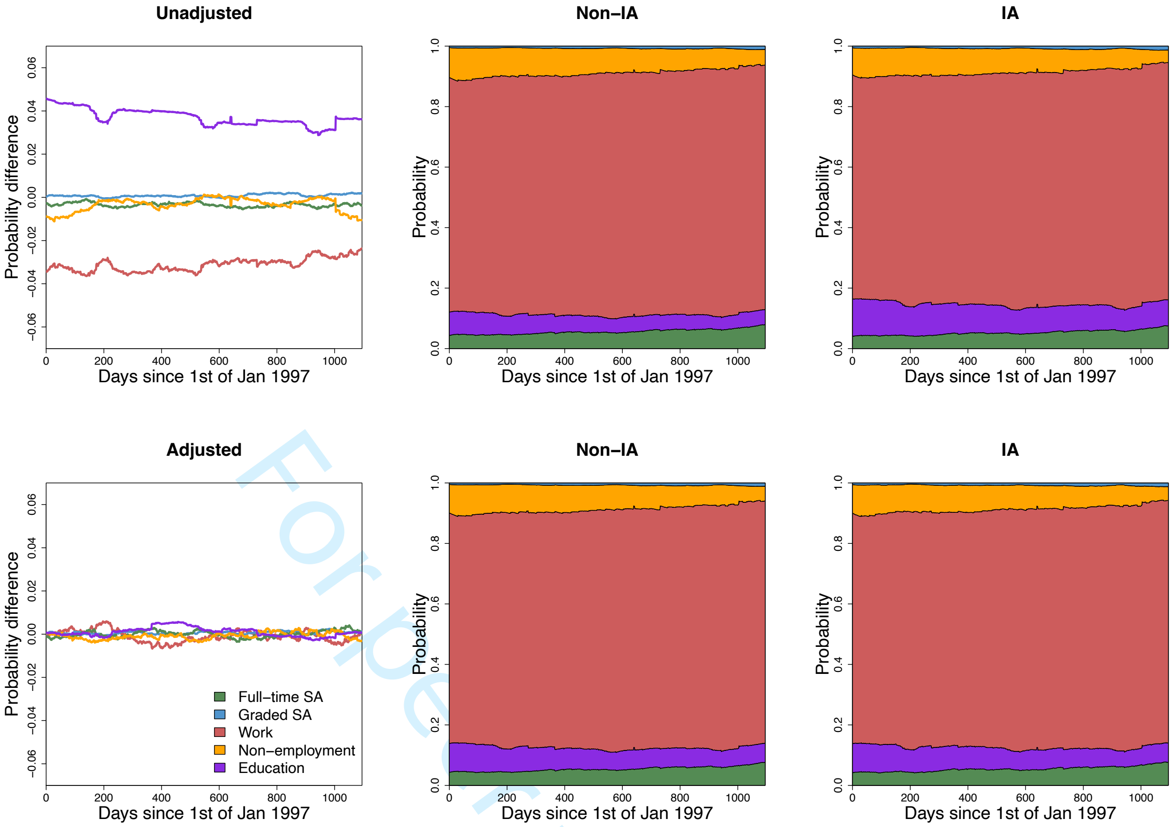

Supplementary Figure 3: The left column shows differences in state probabilities, $\hat{\boldsymbol{\pi}}^1(t) - \hat{\boldsymbol{\pi}}^0(t)$, where superscript 1 denotes having the Inclusive Working Life Agreement (IA) and superscript 0 denotes no IA. The middle and right column show state occupation probabilities for no IA and IA respectively. The top row shows unadjusted estimates, while the bottom row shows estimates adjusted for confounding using inverse probability of treatment (IPT) weighting.

For peer review only

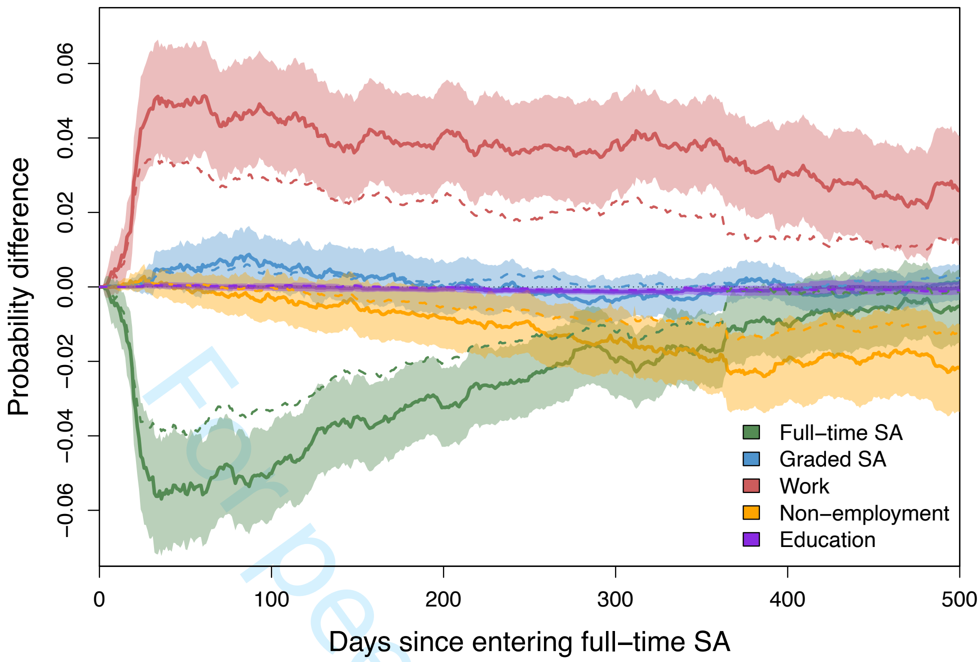

Supplementary Figure 4: Difference in estimated state probabilities, $\hat{\boldsymbol{\pi}}^1(t) - \hat{\boldsymbol{\pi}}^0(t)$, after entering long-term full-time sickness absence (SA), where superscript 1 denotes having the Inclusive Working Life Agreement (IA) and superscript 0 denotes not having IA. Estimates are adjusted for confounding using inverse probability of treatment (IPT) weighting. Faded areas are 95 % confidence intervals based on 1000 bootstrap samples. Full-drawn lines are estimates for the limited selection of industries, wholesale and retail, construction, manufacturing, commercial services and transport and storage, while the stippled lines show the estimates from the primary analysis (all industries).

For peer review only

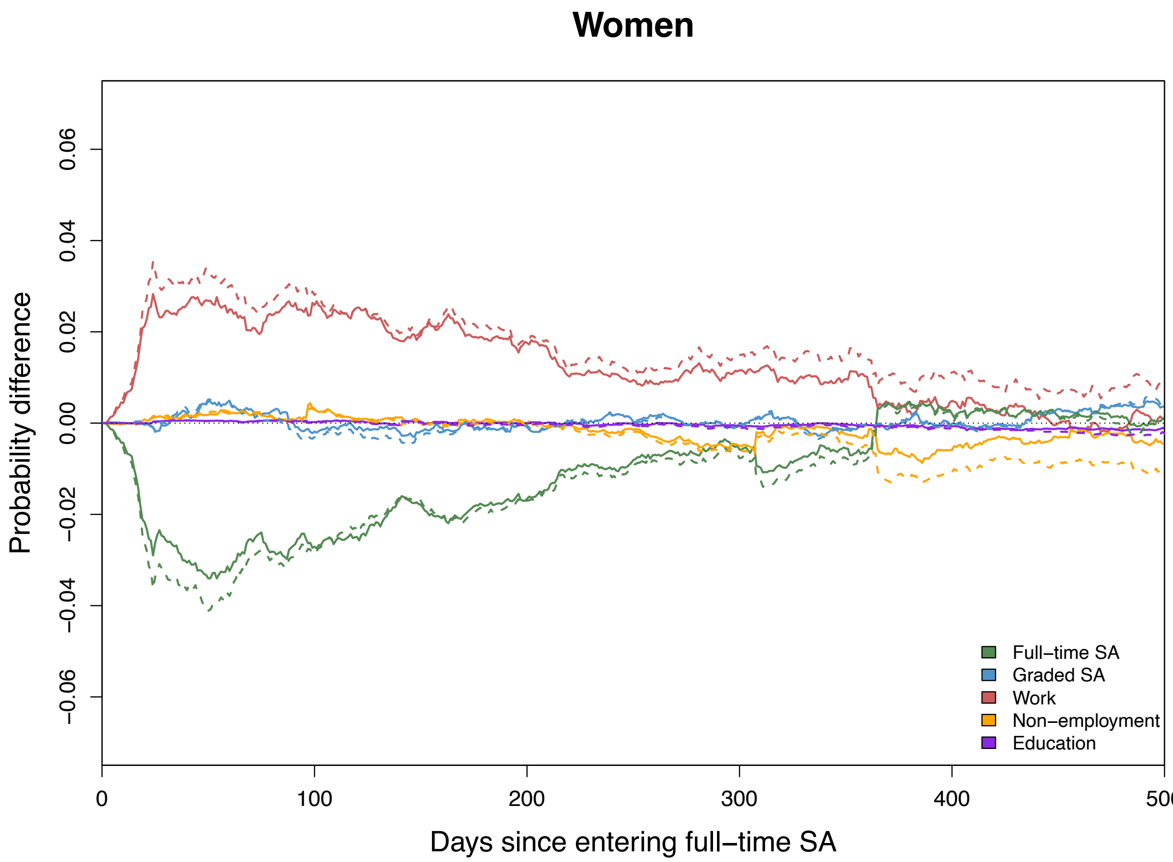

Supplementary Figure 5: Difference in estimated state probabilities, $\hat{\boldsymbol{\pi}}^1(t) - \hat{\boldsymbol{\pi}}^0(t)$, after entering long-term full-time sickness absence (SA), where superscript 1 denotes having the Inclusive Working Life Agreement (IA) and superscript 0 denotes not having IA. Estimates are adjusted for confounding using inverse probability of treatment (IPT) weighting. Faded areas are 95 % confidence intervals based on 1000 bootstrap samples. Full-drawn lines are estimates for all the women in the study population, while the stippled lines show updated estimates after removing the 20,355 women with initial SA diagnosis related to pregnancy, childbearing and family planning.

For peer review only - http://bmjopen.bmj.com/site/about/guidelines.xhtml

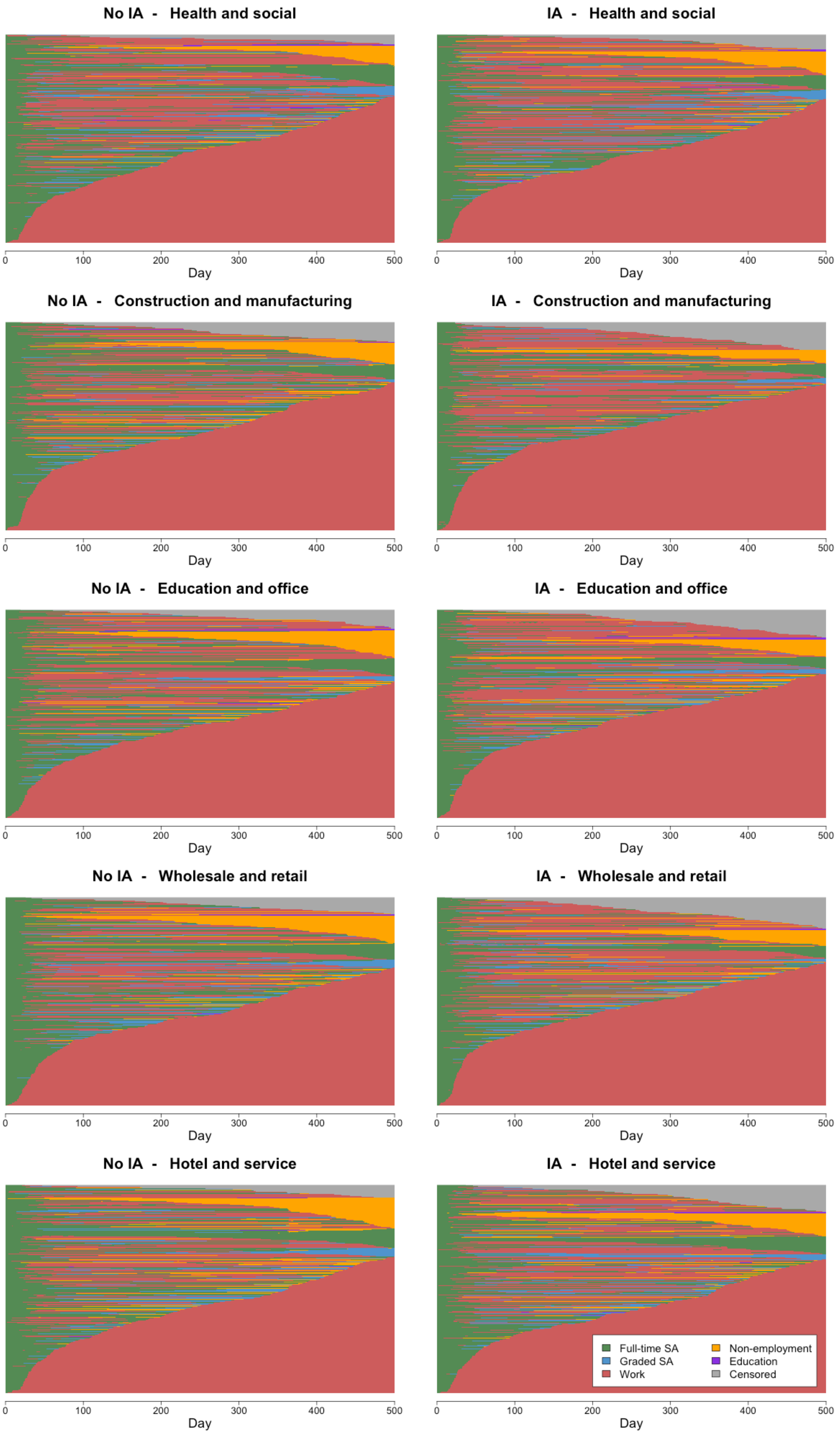

Supplementary Figure 6: State histories for 500 randomly sampled individuals within each group of industry. The sequences are ordered bottom up based the state occupied on the last observation day (day 500) and backwards on this state order: work, partial sick-leave, full-time sick-leave, non-employment, death, education and censoring.

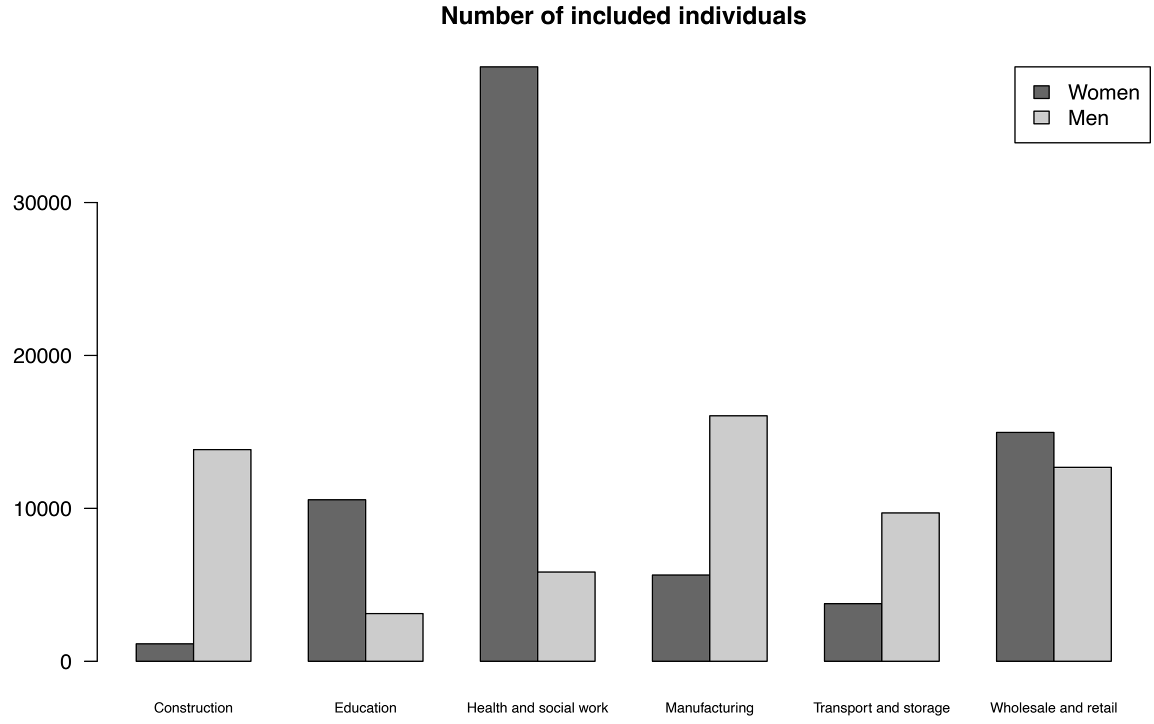

Supplementary Figure 7: Number of included individuals working in the six most represented industries of the study population.

view only

STROBE Statement—Checklist of items that should be included in reports of ***cohort studies***

	Item No	Recommendation	Page No
**Title and abstract**	1	(*a*) Indicate the study's design with a commonly used term in the title or the abstract<br>(*b*) Provide in the abstract an informative and balanced summary of what was done and what was found	a) 1<br>b) 1
**Introduction**			
Background/rationale	2	Explain the scientific background and rationale for the investigation being reported	1-2
Objectives	3	State specific objectives, including any prespecified hypotheses	1-2
**Methods**			
Study design	4	Present key elements of study design early in the paper	2
Setting	5	Describe the setting, locations, and relevant dates, including periods of recruitment, exposure, follow-up, and data collection	2-3
Participants	6	(*a*) Give the eligibility criteria, and the sources and methods of selection of participants. Describe methods of follow-up<br>(*b*) For matched studies, give matching criteria and number of exposed and unexposed	a) 2
Variables	7	Clearly define all outcomes, exposures, predictors, potential confounders, and effect modifiers. Give diagnostic criteria, if applicable	2-3
Data sources/ measurement	8*	For each variable of interest, give sources of data and details of methods of assessment (measurement). Describe comparability of assessment methods if there is more than one group	2-3
Bias	9	Describe any efforts to address potential sources of bias	2-3
Study size	10	Explain how the study size was arrived at	2
Quantitative variables	11	Explain how quantitative variables were handled in the analyses. If applicable, describe which groupings were chosen and why	2-3
Statistical methods	12	(*a*) Describe all statistical methods, including those used to control for confounding<br>(*b*) Describe any methods used to examine subgroups and interactions<br>(*c*) Explain how missing data were addressed<br>(*d*) If applicable, explain how loss to follow-up was addressed<br>(*e*) Describe any sensitivity analyses	a) 3-4, S1-2<br>b) 3<br>c) 2<br>e) 3
**Results**			
Participants	13*	(a) Report numbers of individuals at each stage of study—eg numbers potentially eligible, examined for eligibility, confirmed eligible, included in the study, completing follow-up, and analysed<br>(b) Give reasons for non-participation at each stage<br>(c) Consider use of a flow diagram	a) 2<br>b) 2
Descriptive data	14*	(a) Give characteristics of study participants (eg demographic, clinical, social) and information on exposures and potential confounders<br>(b) Indicate number of participants with missing data for each variable of interest<br>(c) Summarise follow-up time (eg, average and total amount)	a) 4<br>c) 4,S4
Outcome data	15*	Report numbers of outcome events or summary measures over time	sup4

For peer review only

For peer review only - http://bmjopen.bmj.com/site/about/guidelines.xhtml

Main results	16	(*a*) Give unadjusted estimates and, if applicable, confounder-adjusted estimates and their precision (eg, 95% confidence interval). Make clear which confounders were adjusted for and why they were included<br>(*b*) Report category boundaries when continuous variables were categorized<br>(*c*) If relevant, consider translating estimates of relative risk into absolute risk for a meaningful time period	a) 4,<br>b) 4
Other analyses	17	Report other analyses done—eg analyses of subgroups and interactions, and sensitivity analyses	6-7<br>S2-7
**Discussion**			
Key results	18	Summarise key results with reference to study objectives	7-8
Limitations	19	Discuss limitations of the study, taking into account sources of potential bias or imprecision. Discuss both direction and magnitude of any potential bias	8
Interpretation	20	Give a cautious overall interpretation of results considering objectives, limitations, multiplicity of analyses, results from similar studies, and other relevant evidence	8
Generalisability	21	Discuss the generalisability (external validity) of the study results	8
**Other information**			
Funding	22	Give the source of funding and the role of the funders for the present study and, if applicable, for the original study on which the present article is based	8

*Give information separately for exposed and unexposed groups.

Note: An Explanation and Elaboration article discusses each checklist item and gives methodological background and published examples of transparent reporting. The STROBE checklist is best used in conjunction with this article (freely available on the Web sites of PLoS Medicine at http://www.plosmedicine.org/, Annals of Internal Medicine at http://www.annals.org/, and Epidemiology at http://www.epidem.com/). Information on the STROBE Initiative is available at http://www.strobe-statement.org.

For peer review only

For peer review only - http://bmjopen.bmj.com/site/about/guidelines.xhtml
